# Estimating the long-term effects of mass screening for latent and active tuberculosis in the Marshall Islands

**DOI:** 10.1093/ije/dyac045

**Published:** 2022-03-22

**Authors:** Romain Ragonnet, Bridget M Williams, Angela Largen, Joaquin Nasa, Tom Jack, Mailynn K Langinlur, Eunyoung Ko, Kalpeshsinh Rahevar, Tauhid Islam, Justin T Denholm, Ben J Marais, Guy B Marks, Emma S McBryde, James M Trauer

**Affiliations:** School of Public Health and Preventive Medicine, Faculty of Medicine, Nursing and Health Sciences, Monash University, Melbourne, VIC, Australia; School of Public Health and Preventive Medicine, Faculty of Medicine, Nursing and Health Sciences, Monash University, Melbourne, VIC, Australia; Hawaii Department of Health, Tuberculosis Control Branch, Honolulu, HI, USA; Ministry of Health and Human Services, Majuro, Marshall Islands; Ministry of Health and Human Services, Majuro, Marshall Islands; Ministry of Health and Human Services, Majuro, Marshall Islands; WHO Country Liaison Office, Micronesia, Department of Health and Social Affairs, Palikir, Pohnpei, Federated States of Micronesia; World Health Organization Regional Office for the Western Pacific (WHO WPRO), Manila, Philippines; World Health Organization Regional Office for the Western Pacific (WHO WPRO), Manila, Philippines; Victorian Tuberculosis Program, Royal Melbourne Hospital, Melbourne, VIC, Australia; Sydney Institute for Infectious Diseases (Sydney ID) and the WHO Collaborating Centre for Tuberculosis, University of Sydney, Westmead, NSW, Australia; South Western Sydney Clinical School, University of New South Wales, Liverpool, NSW, Australia; Australian Institute of Tropical Health and Medicine, James Cook University, Douglas, QLD, Australia; School of Public Health and Preventive Medicine, Faculty of Medicine, Nursing and Health Sciences, Monash University, Melbourne, VIC, Australia

**Keywords:** Mycobacterium tuberculosis infection, active case-finding, latent tuberculosis infection, mass screening, post-exposure prevention

## Abstract

**Background:**

Ambitious population-based screening programmes for latent and active tuberculosis (TB) were implemented in the Republic of the Marshall Islands in 2017 and 2018.

**Methods:**

We used a transmission dynamic model of TB informed by local data to capture the Marshall Islands epidemic’s historical dynamics. We then used the model to project the future epidemic trajectory following the active screening interventions, as well as considering a counterfactual scenario with no intervention. We also simulated future scenarios including periodic interventions similar to those previously implemented, to assess their ability to reach the End TB Strategy targets and TB pre-elimination in the Marshall Islands.

**Results:**

The screening activities conducted in 2017 and 2018 were estimated to have reduced TB incidence and mortality by around one-third in 2020, and are predicted to achieve the End TB Strategy milestone of 50% incidence reduction by 2025 compared with 2015. Screening interventions had a considerably greater impact when latent TB screening and treatment were included, compared with active case finding alone. Such combined programmes implemented at the national level could achieve TB pre-elimination around 2040 if repeated every 2 years.

**Conclusions:**

Our model suggests that it would be possible to achieve TB pre-elimination by 2040 in the Marshall Islands through frequent repetition of the same interventions as those already implemented in the country. It also highlights the importance of including latent infection testing in active screening activities.

Key Messages

- The community-wide active case-finding interventions conducted in the Marshall Islands in 2017 and 2018 will cause significant tuberculosis (TB) incidence and mortality reductions over the following decades.
- These reductions will be considerably more significant in the regions that included latent TB infection screening in their programme.
- TB pre-elimination could be achieved by 2040 in the Marshall Islands through periodic repetition of the same interventions as those already implemented in the country.
- It is possible to rapidly drive TB towards elimination in high-TB-burden settings using currently available TB control tools.
- This can be achieved with ambitious interventions that target latent TB infection in addition to active TB.

## Introduction

Every year, around 10 million people develop tuberculosis (TB) disease globally.^1^ The causative agent of TB (*Mycobacterium tuberculosis*, *M.tb*) was first identified in 1882—and yet more than 130 years later, TB is estimated to be the world’s leading cause of death from a single infectious agent.^1^ The availability of effective vaccines and treatments has not been sufficient to eliminate TB. Consequently, global disease elimination is not expected in the coming decades without dramatic changes to current TB control approaches, especially in high-incidence countries.

Improving case detection is a promising way to progress the global TB response, because millions of diseased individuals are undetected.^1^ Active case-finding (ACF) activities can identify significant numbers of individuals with active disease who may not have been detected otherwise or may only have been detected after substantial delays. Recent large-scale randomized trials of ACF interventions in high-burden settings have demonstrated the efficacy of these approaches and generated hope for dramatically improved control by reducing the pool of infectious individuals.^2^^,^^3^ However, the ability of ACF to achieve substantial TB burden reductions is likely to vary by setting, such that contextualized studies are needed to assess its impact in different locations.

Screening and treatment of latent TB infection (LTBI) can also be included in these ambitious programmes to prevent future TB reactivation in infected individuals. Large-scale cluster-randomized trials of preventive treatment were conducted in the late 1950s in Alaska, Greenland and Tunisia, all demonstrating significant TB incidence reductions.^4–6^ However, a more recent trial conducted in a South African mining population with a high prevalence of HIV was not successful in reducing TB burden.^7^ This demonstrates that the effectiveness of prophylaxis programmes depends on the characteristics of the population in which the intervention is implemented,^8^ and that it is difficult to anticipate the effects of future interventions by extrapolating observations made in other settings.

Ambitious population-level screening programs were recently completed in the Republic of the Marshall Islands (hereinafter referred to as ‘the Marshall Islands’), an archipelago nation in the western Pacific. The Marshall Islands is a high-TB-burden country with an estimated TB incidence approaching 500 cases per 100 000 population per year in 2019, according to the World Health Organization (WHO).^9^ The screening activities were conducted in the two most populous island groups of the Marshall Islands—Ebeye and Majuro—together representing nearly three-quarters of the national population. In 2017, an initial ACF intervention aimed to screen the entire adult population (those aged 15 years or older) of Ebeye Island for active TB disease and to treat those with suspected disease. The following year, an even more comprehensive intervention was conducted on Majuro Atoll to screen individuals of all ages for both latent and active TB, and provided appropriate treatment for both these conditions. These programmes have enabled the identification and treatment of a large number of infected individuals. It is now critical to estimate the long-term effects of these interventions and to identify effective follow-up approaches that would sustain significant TB incidence reductions in the Marshall Islands.

In this modelling study, we incorporated local data collected during the interventions into a transmission dynamic model of TB capturing important features of the local TB epidemic, including time-variant case detection, BCG vaccination and type-2 diabetes. We then used the model to assess whether the Marshall Islands could achieve the End TB Strategy targets and pre-elimination under the current strategy, as well as under various future intervention scenarios.

## Methods

### Overall approach

We used a deterministic compartmental model to simulate *M.tb* transmission in the Marshall Islands, using a similar approach to previously published studies.^10–12^ After calibrating the model to local data using Bayesian techniques, we projected the long-term effect of the large-scale LTBI and TB screening activities undertaken in 2017 and 2018. We then simulated periodic interventions similar to those previously implemented and covering the entire country at regular intervals. We assessed prospects for reaching the End TB Strategy milestones and targets and the pre-elimination incidence threshold (see Table 1).

**Table 1:** End TB Strategy targets and milestones and pre-elimination threshold

Target or milestone	Definition
TB incidence	
2025 milestone^13^	50% reduction between 2015 and 2025
2035 target^13^	90% reduction between 2015 and 2035
Pre-elimination threshold^14^	TB incidence below 1 case per 100 000 population per year
TB mortality	
2025 milestone^13^	75% reduction between 2015 and 2025
2035 target^13^	95% reduction between 2015 and 2035

TB, tuberculosis.

### Tuberculosis model

**Figure 1:**
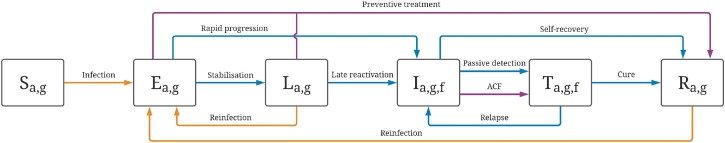
Illustration of the model structure. Boxes represent the different compartments types: susceptible (S), early latent (E), late latent (L), infectious (I), on treatment (T) and recovered (R). The subscripts indicate whether compartments are stratified by age (a), geography (g) and form of tuberculosis (f). Blue and orange arrows represent progression flows and transmission flows, respectively. The flows associated with the modelled interventions are shown in purple. ACF, active case finding

The base model consisted of six compartments representing particular clinical statuses regarding *M.tb* infection or disease (Figure 1). LTBI was modelled using two sequential compartments (*E* and *L*) to capture the declining risk of disease progression over time from infection.^15^ Individuals progressing to active TB (*I*) were classified based on their form of disease: smear-positive pulmonary TB, smear-negative pulmonary TB, and extrapulmonary TB. The simulated population was stratified by age and location (Majuro Atoll, Ebeye Island, and all other islands of the Marshall Islands) and we allowed inter-island mixing. In the base-case analysis we assumed that 95% of interpersonal contacts occur between individuals of the same island group, whereas this proportion was reduced to 80% in a sensitivity analysis. The model captured the effect of type 2 diabetes on the risk of TB progression, accounting for the increase in diabetes prevalence over recent decades. BCG vaccination was incorporated through a time-variant and age-specific process reducing the risk of infection (base-case analysis) or reducing the severity of TB (sensitivity analysis). More details about the model are available in the Supplementary Material (available as Supplementary data at *IJE* online).

We used local programmatic data to inform the simulated detection and treatment processes. Passive case detection of TB was assumed to commence from around 1950 and to increase progressively with time, to reflect improvements in TB control. Two different passive screening scale-up profiles were considered: one including a rapid screening rate increase after 1980 (base case) and one assuming that passive detection has remained constant since 1980 (sensitivity analysis). Our previously published estimates were used to inform the natural history of TB^16^ and the progression rates from latent to active TB.^15^ We fitted model parameters using local data on population size, TB prevalence, LTBI prevalence and TB notifications, while considering uncertainty around the most critical model parameters (Table 2). The code used to implement the model is publicly available on Github [https://github.com/monash-emu/AuTuMN/releases/tag/TB_Marshall_Islands_Int_J_Epidemiol].

**Table 2:** Model parameters

Parameter	Value or uncertainty range	Source
**Population characteristics**		
Targeted total population size (2011, all Marshall Islands)	53 158	2011 National Census
Population size at the start of simulation (1800)	200–1000	Fitted
Population proportions in Majuro, Ebeye and other islands (2011)	52%/20%/28%	2011 National Census
Proportion of contacts that occur with individuals from the same geographical group	95% (80% in sensitivity analysis)	Assumption. Remainder of contacts distributed evenly between the two other locations (2.5% each in base-case analysis, 10% each in sensitivity analysis)
Crude birth rate	Time-variant	United Nations Population Division data for the Federated States of Micronesia
All-cause mortality rate	Age-specific and time-variant	United Nations Population Division
Type 2 diabetes prevalence	Age-specific and time-variant	Assumed diabetes proportions in 2020 based on the age-adjusted prevalence reported by the International Diabetes Federation Diabetes Atlas^17^: 0-4: 1% 5-14: 5% 15-34: 20% 35-49: 40% 50+: 70%
***M.tb* infection and TB disease**		
Transmission scaling factor	0.002–0.010	Fitted
Relative infectiousness (smear-positive/smear-negative/extrapulmonary TB)	1/0.25/0	^18^ ^,^ ^19^
Relative infectiousness by age	Progressive increase through childhood (Supplementary Material, available as Supplementary data at *IJE* online)	^20^ ^,^ ^21^
Relative infectiousness during treatment (ref. untreated TB)	0.08	Based on the assumption that patients are infectious for the first 2 weeks of a 6-month regimen
Rate of stabilization from early to late latency (age 0-4/5-14/15+)	4.4/4.4/2 per year	^15^
Rate of rapid progression to active TB (age 0-4/5-14/15+)	2.4/2/0.1 per year	^15^
Rate of late reactivation (age 0-4/5-14/15+)	7e^-9^/2.3e^-3^/1.2e^-3^ per year	^15^
Uncertainty multiplier for the rates of TB progression	0.5–2	Fitted^15^
Relative risk of TB progression for individuals with type 2 diabetes	2–5	^22^
Proportion of pulmonary TB among incident TB	85%	Adjusted to replicate observed prevalence proportions by form of TB
Proportion of smear-positive TB among incident pulmonary TB	75%	Adjusted to replicate observed prevalence proportions by form of TB
Rate of self-recovery (smear-positive TB/other forms of TB)	0.18—0.29/0.07—0.21 per year	^16^
Rate of TB-specific mortality (smear-positive TB/other forms of TB)	0.34—0.45/0.017—0.035 per year	^16^
Relative risk of reinfection while latently infected (ref. infection-naive)	0.2–0.5	^23^
Relative risk of reinfection after recovery (ref. infection-naive)	0.2–1	^23^
**TB control**		
BCG vaccination coverage	Time-variant	Global Health Observatory data repository (WHO)
Reduced susceptibility to infection due to BCG vaccination	Age-specific	^24^ ^,^ ^25^
Passive TB screening rate	Varies with time and location	Fitted (see Supplementary Material, available as Supplementary data at *IJE* online)
TB screening sensitivity (smear-positive/smear-negative/extrapulmonary TB)	100%/70%/50%	Assumption^a^
Treatment success rate	Time-variant	WHO
Proportion of death among non-successful treatment	20%	WHO
Average TB treatment duration	6 months	Assumption
Active case finding rate	Varies with time, location and scenario	–
LTBI screening rate	Varies with time, location and scenario	–
LTBI screening sensitivity	75% (varied in sensitivity analysis)	^26^
Preventive treatment efficacy (intention-to-treat)	75–85%	Based on completion rate during intervention
Relative improvement in passive screening rate following interventions	0–50%	Assumption based on discussions with the national programme

The ranges presented for the fitted parameters correspond to the ranges used to inform the prior distributions in the adaptive Metropolis algorithm. The values of the parameters that are time-variant and/or age-specific are presented in the Supplementary Material (available as Supplementary data at *IJE* online).

*M.tb*, *Mycobacterium tuberculosis*; TB, tuberculosis; LTBI, latent tuberculosis infection; BCG, Bacille Calmette-Guerin; WHO, World Health Organization.

a These parameters are multiplied by the fitted screening rate parameter, such that their absolute values are less significant than the relative values between the different forms of TB.

### Modelled interventions

Latently infected individuals were assumed to transition to the recovered compartment on completion of LTBI treatment, at a rate defined as the product of a time-variant LTBI screening rate, the LTBI test's sensitivity (varied in a sensitivity analysis) and the efficacy of preventive treatment (varied during calibration). The effect of ACF was modelled using a time-variant screening rate multiplied by the sensitivity of the diagnostic test used during the ACF intervention, and was assumed to move individuals from the undetected disease compartment to the treatment compartment (Figure 1). Screening rates were parameterized such that the modelled proportions of screened individuals by the end of the interventions matched those measured in the field. To capture the future effect of increased TB awareness due to the large-scale community interventions, we increased the rate of passive screening of active TB from 2018―the endpoint of the completed intervention―considering relative improvements in the screening rate ranging from zero to 50%. Whereas the base-case analysis assumed no LTBI importation through migration, we evaluated the impact of imported LTBI in a sensitivity analysis considering an LTBI prevalence of up to 40% among immigrants and assuming an immigration rate of 300 people per year (estimated from the 2011 census). We assigned 10% of imported LTBI cases to the early latent compartment (*E*) and the remaining 90% to the late latent compartment (*L*). This is approximately equivalent to assuming that 10% of migrants with LTBI were infected in the 6 months preceding migration.

We also modelled scenarios where the community-wide interventions would be repeated periodically at the national level, every 2, 5 or 10 years, starting from 2021. We assumed a similar screening rate to that of the intervention conducted in Majuro in 2018. Finally, we estimated the numbers of TB episodes and TB deaths averted by 2050 attributable to the screening interventions under the different scenarios, and we compared these outputs with the estimated total number of serious adverse events (SAEs) due to preventive treatment. In this analysis we considered an SAE probability of 1.6% among individuals completing preventive treatment with 3 months of combined rifapentine and isoniazid (3HP) treatment,^27^ as was used during the Majuro intervention in 2018.

## Results

**Figure 2:**
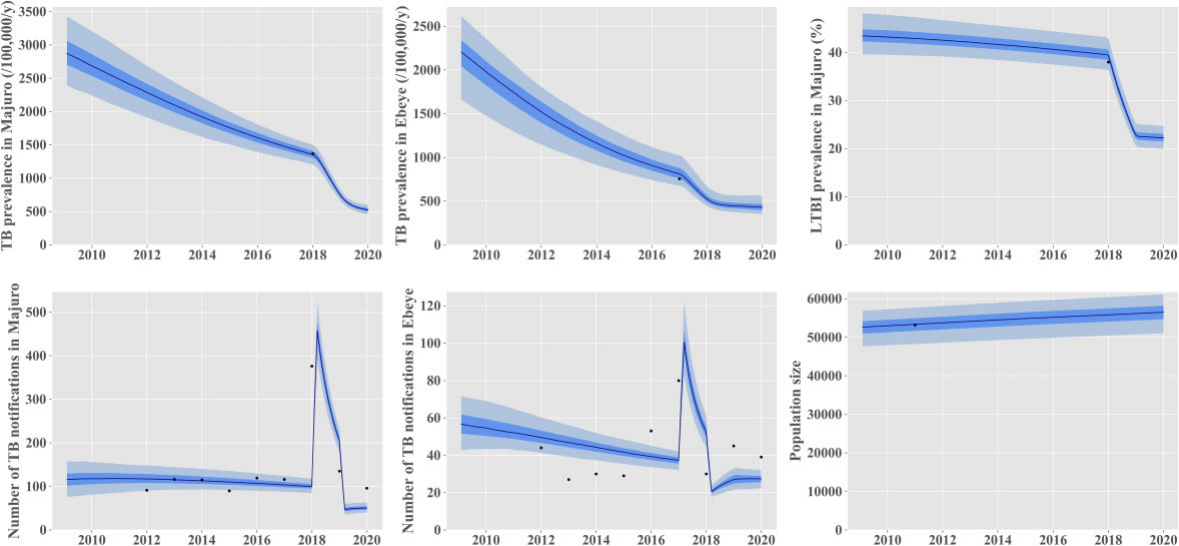
Comparison between model outputs and local data for the calibration targets. The black dots represent local empirical data. The model predictions are represented in blue as median (solid line), interquartile credible interval (dark shade) and 95% central credible interval (light blue shade). The effect of the 2017-18 interventions was included in these projections. TB, tuberculosis

**Figure 3:**
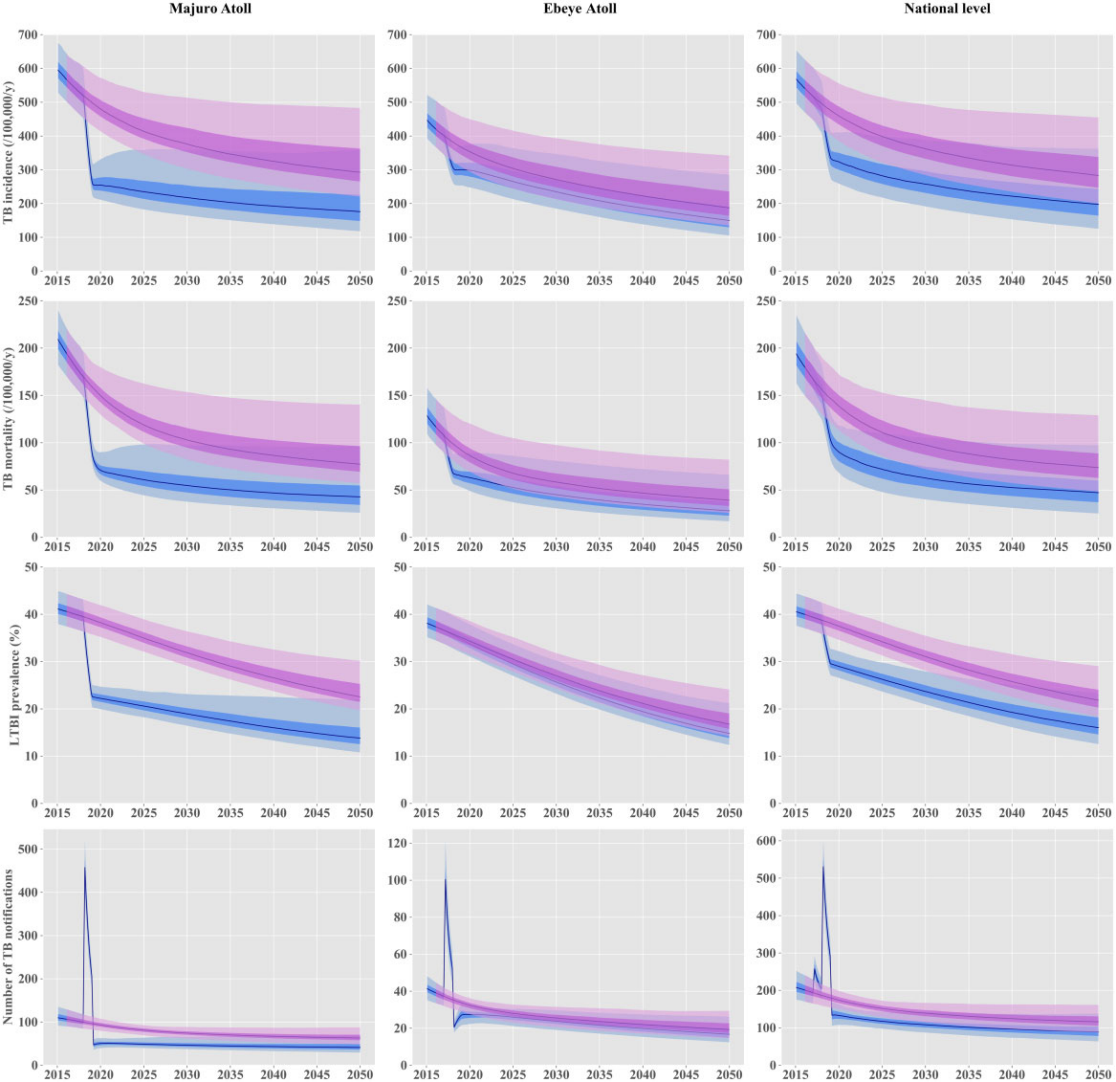
Projected effect of the active screening interventions implemented in 2017 and 2018. The solid lines represent the median estimates. The shaded areas show the interquartile credible intervals (dark shade) and 95% credible intervals (light shade) projected in the absence of any intervention (pink) and under a scenario including the interventions implemented in 2017-18 in Majuro and Ebeye (blue). TB, tuberculosis; LTBI, latent tuberculosis infection

Figure 2 presents the base-case model fits to local data. Our model suggests that TB incidence has been declining steadily over the past 20 years, reflecting improvements in case detection and treatment as well as increasing BCG vaccination coverage over time (Figure 2; and Supplementary Figure S3, available as Supplementary data at *IJE* online). The epidemic decline was projected to be continued until at least 2050, even in the absence of the 2017–18 interventions (Figure 3). Posterior parameter estimates are shown in the Supplementary Table S3 (available as Supplementary data at *IJE* online).

### Effect of the 2017–18 screening interventions

**Figure 4:**
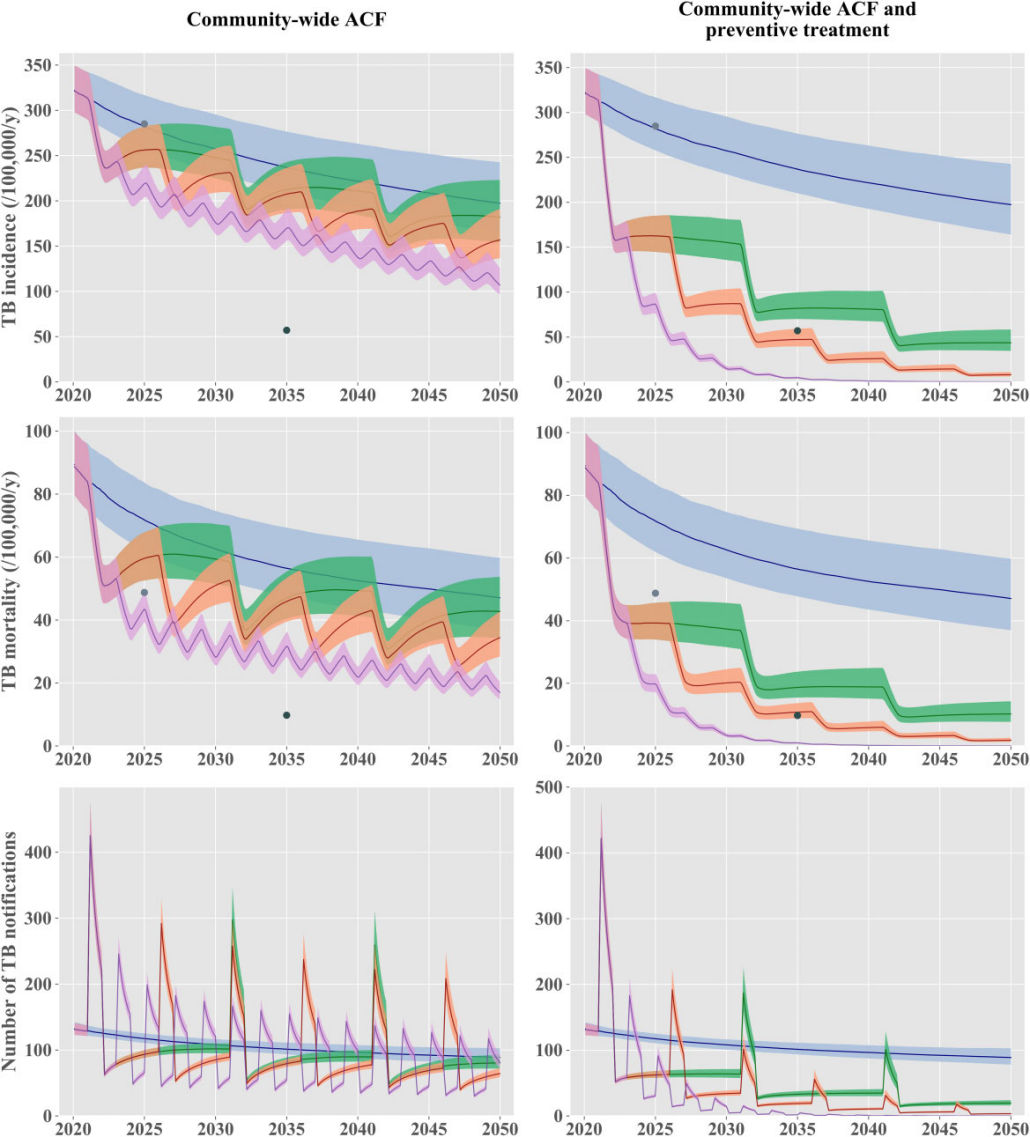
Projected effect of periodic community-wide interventions. The solid lines represent the median estimates, and the shaded areas show the interquartile credible intervals. The ‘status-quo’ scenario is represented in blue in all panels. The left column of panels presents scenarios including nationwide active case finding (ACF) repeated every 2 years (purple) or every 5 years (orange) or every 10 years (green). The right column of panels presents nationwide ACF scenarios combined with mass latent infection screening and treatment, repeated every 2 years (purple) or 5 years (red). The light and dark grey dots show the 2025 milestones and the 2035 targets, respectively, according to the End TB Strategy. TB, tuberculosis; ACF, active case finding

Figure 3 presents the future projected epidemic trajectories with and without the implementation of the active screening interventions. At the national level, we estimated that the 2017–18 interventions had reduced TB incidence by ∼30% in 2020 compared with the counterfactual no-intervention scenario. We estimated that the 2020 TB incidence would have been 459 [95% credible interval (CrI) 385–556] per 100 000 population per year without intervention and 323 (95% CrI 259–411) per 100 000 population per year when including the 2017–18 interventions. TB mortality was predicted to have decreased by around 35% from 139 (95% CrI 113–172) to 90 (95% CrI 68–118) per 100 000 population per year in 2020 due to the screening interventions. The community-wide interventions had a considerably greater effect on the epidemic in Majuro than in Ebeye. In Majuro, the interventions achieved an estimated 47% reduction in the local TB incidence and mortality in 2020, compared with the counterfactual no-intervention scenario, with effects sustained until at least 2050 with no further intervention. In Ebeye, the estimated incidence reduction induced by the screening activities reached 20% in 2050, compared with the counterfactual no-intervention scenario. If current programmatic conditions were continued with no future ACF interventions, and assuming enhanced TB awareness following the interventions, we predicted that the Marshall Islands could achieve the 2025 End TB incidence milestone (Figure 4). However, the country would fall short of reaching any TB mortality targets and the 2035 TB incidence target.

### Impact of repeated screening interventions every two, five or ten years

The projections considering repeated interventions at the country level every 2, 5 or 10 years are presented in Figure 4. If LTBI screening were not included in the ACF programmes, we predicted that the 2035 End TB Strategy targets would not be reached, regardless of the intervention frequency considered. Combining ACF with LTBI screening and treatment would have considerably greater impacts on future TB burden. We estimated that all the End TB Strategy targets could be reached if this intervention was repeated every 2 or 5 years. However, the Marshall Islands would fall short of reaching the incidence and mortality targets in 2035 under the scenario considering a 10-year screening cycle. Results similar to those presented in Figure 4 are shown on a log-scale in Supplementary Figure S13 (available as Supplementary data at *IJE* online), displaying the pre-elimination threshold defined as a TB incidence rate of one case per 100 000 population per year.^14^ This showed that the Marshall Islands could achieve pre-elimination around 2040 with community-wide screening of latent and active TB repeated every 2 years at the country level. The 5-year and 10-year screening cycles were not predicted to reach pre-elimination by 2050, although both scenarios were estimated to reduce TB incidence to below 100 per 100 000 population per year by 2035.

### Sensitivity analyses

The results of our analysis considering different assumptions for the future trend of diabetes prevalence are shown in Supplementary Figure S4 (available as Supplementary data at *IJE* online). This analysis included the interventions previously conducted in Majuro and Ebeye and assumed that TB control would remain similar to the current programmatic situation until 2050. If diabetes prevalence increased by 20% by 2050, the predicted TB incidence in 2050 would be 243 (95% CrI 149–454) per 100 000 population per year. In contrast, we estimated that a 20% decline in diabetes prevalence would be associated with a further decrease in TB incidence to 158 (95% CrI 102–284) per 100 000 population per year in 2050.

**Figure 5:**
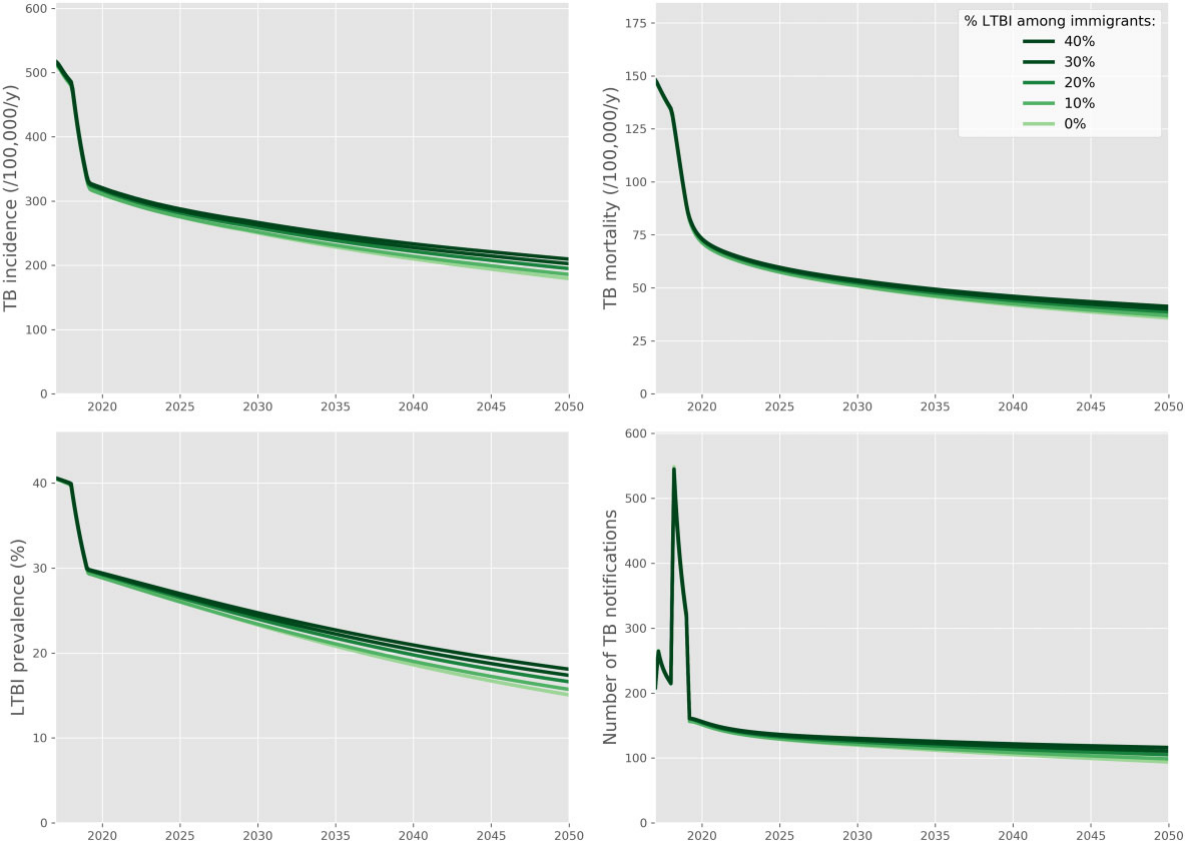
Results of the sensitivity analysis considering different rates of latent tuberculosis importation in the future. This analysis considered the ‘status-quo’ scenario including the 2017-18 interventions. We assumed an immigration rate of 300 per year. We used the maximum likelihood estimates obtained during calibration (i.e. the best-fitted run) to inform the parameters used in this analysis. TB, tuberculosis; LTBI, latent tuberculosis infection

Our sensitivity analysis considering future importation of LTBI cases through immigration suggests moderate impacts on future TB burden from LTBI screening among immigrants (Figure 5). We estimated that TB incidence in 2050 would be 17% higher if 40% of the immigrating population had LTBI compared with what it would be without imported LTBI. Other sensitivity analyses showed that our findings would remain virtually unchanged under increased inter-island mixing (Supplementary Figures S11 and S12, available as Supplementary data at *IJE* online) or if BCG reduced TB severity rather than the risk of *M.tb* infection (Supplementary Figures S6 and S7, available as Supplementary data at *IJE* online). Considering different levels of LTBI screening sensitivity was not found to affect the conclusions regarding the long-term impact of the 2017–18 interventions (Supplementary Figure S5, available as Supplementary data at *IJE* online).

Finally, assuming that the passive screening rate has been constant after 1980 resulted in significantly more pessimistic projections of future TB burden across all scenarios considered, compared with the base-case analysis assuming increasing detection rates after 1980. However, the estimated TB burden reductions achieved by the 2017–18 interventions remained very similar to what was estimated in the base-case analysis, with a 29% TB incidence reduction and a 36% TB mortality reduction in 2020 compared with the counterfactual no-intervention scenario.

### Comparing the benefits and the risks associated with the interventions

**Figure 6:**
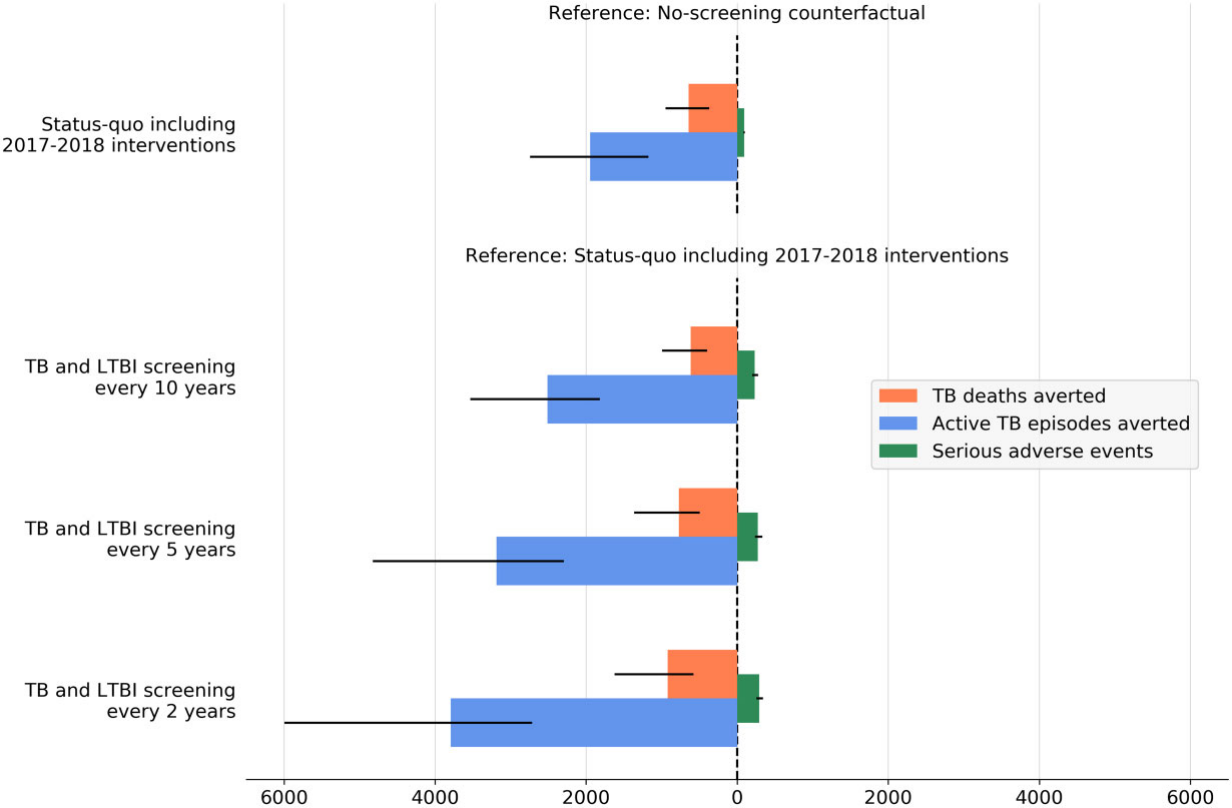
Estimated tuberculosis disease episodes and tuberculosis deaths averted by active screening interventions, compared with serious adverse effects induced by preventive treatment. Coloured bars show the median estimates and the thin black bars indicate the 95% credible intervals. Numbers of tuberculosis disease episodes, tuberculosis deaths and serious adverse events were cumulated over the period 2017-50. TB, tuberculosis; LTBI, latent tuberculosis infection

Our evaluation of the benefit-risk of active screening including LTBI treatment suggests that the 2017–18 interventions will have overwhelming benefits compared with the risk of SAEs induced by preventive treatment (Figure 6; and Supplementary Table S5, available as Supplementary data at *IJE* online). We estimated that by 2050, the existing interventions will have averted 1948 (95% CrI 1173–2742) TB disease episodes and 643 (95% CrI 370–949) TB deaths, whereas the total number of SAEs was estimated at 93 (95% CrI 84–102). Similarly, repeating active screening in the future was estimated to yield numbers of cases and deaths averted which were significantly higher than the number of SAEs, regardless of the screening frequency considered.

## Discussion

In 2017 and 2018, the Marshall Islands conducted unprecedented TB and LTBI screening activities. These ambitious community-led interventions were supported by exceptional efforts from local and external stakeholders and by volunteers. Success has already been demonstrated through the large number of detected individuals with latent or active TB who completed treatment. Our modelling projections now suggest that these efforts will have considerable effects on the local TB epidemics' long-term trajectory. We also estimate that periodic use of interventions such as those implemented in the Marshall Islands could achieve pre-elimination goals over the coming decades if the screening efforts were repeated every second year.

ACF is a high-intensity, high-resource effort. Going beyond TB case-finding by adding treatment of LTBI increases the cost and the time taken to screen a population, but our model demonstrates a considerably higher impact when both active and latent TB are addressed together. This suggests that considerable resources are likely to be required to yield the dramatic impacts on TB burden we projected. However, we also show that impacts could be sustained in the long term and that the Marshall Islands could achieve pre-elimination through such interventions. This means that substantial returns on investment are anticipated as many TB cases and deaths will be averted over several decades. In addition to the human and societal benefits, these direct health effects also translate into economic savings because of the known catastrophic impact that TB has on individuals’, families’ and countries’ finances.^28^ Our results, therefore, highlight the importance of adopting a long-term vision when planning TB control and when funding surveys of TB response.

Our analysis has important implications for future TB control in the Marshall Islands. The critical importance of reducing the latent infection pool's size has been demonstrated by previous modelling works,^29^ including some that suggested that even if transmission ceased from 2015 onwards, this would be insufficient to achieve the End TB Strategy targets globally.^30^ In the present analysis, we demonstrate that these goals could be achieved by using large-scale screening interventions that are realistic, practical and use only existing technologies, given that such interventions have already been implemented in the field. Our study also reinforces the importance of addressing risk factors and comorbidities in addition to TB itself. In particular, we found diabetes to be a key determinant of the TB epidemic's long-term trends in the Marshall Islands context, consistent with findings from other Pacific Island settings.^12^ This calls for multifactorial approaches that combine TB-specific measures with interventions addressing TB risk factors, whether these tools are medical or social. Our results also suggest that screening immigrants for latent TB infection could yield moderate benefits in the Marshall Islands, although the magnitude of the associated burden reduction directly depends on the prevalence of infection among immigrants. Finally, our model suggests a clear pathway to reaching TB pre-elimination in the Marshall Islands. Indeed, we estimated that TB incidence could be brought below one new case per 100 000 population per year by 2040 if the ambitious intervention conducted in Majuro in 2018 could be repeated every 2 years from 2021 at the national level.

Our study's strengths include the fact that our calibration approach was able to capture key disease indicators accurately while incorporating uncertainty around the most fundamental parameters. Furthermore, the model was directly informed by the most relevant data possible since these were directly measured in the field, including through the interventions themselves. These include data on the prevalence of active and latent TB, which are critical to accurately replicating the local TB burden, along with TB notification data that ensured that the historical trends in case detection were captured appropriately. Finally, we conducted simulations using state-of-the-art computing techniques that are publicly available and have been extensively tested and documented, such that our model could be easily reused to assist TB control in the Marshall Islands and other settings.

Limitations of our study include the significant uncertainties that remain in TB epidemiology. In particular, model projections could be refined with improved knowledge about the effect of preventive therapy on the risk of future reinfection, although our model considers a broad range of assumptions regarding this parameter. Similarly, even if recent studies have produced estimates for the rates of progression from latent to active TB,^15^^,^^31^ the rate of late reactivation remains poorly characterized due to the limited data available.^32^ Refining estimates of this parameter would undoubtedly increase the accuracy of predictions related to interventions involving preventive treatment. Second, whereas our model demonstrates the effects of two different ACF strategies in a high-incidence area, it may not capture all impacts of coordinated TB screening efforts. More nuanced impacts to a local or national TB programme could include improvements to TB diagnosis, scaling up efforts to identify close contacts and increasing resources for LTBI identification and management among contacts and other high-risk groups. These programmatic changes are more difficult to quantify in a way that could be incorporated into a model and could also lead to sustainable TB incidence reductions. Third, the model allows interactions between individuals of different island groups, although empirical data were not available to quantify this social mixing process. However, our sensitivity analysis considering alternative inter-island mixing patterns produced the same conclusions as our base-case analysis. Finally, we stress that the projections presented in this study are intended to be primarily relevant to the Marshall Islands context and, given the variability of TB epidemics in different settings, drawing quantitative conclusions for other settings would require dedicated analyses.^33^ In particular, our study did not consider drug-resistant (DR)-TB as its incidence is extremely low in the Marshall Islands. Our model should therefore be adapted to account for this consideration if it were applied to settings where DR-TB is prevalent.

Repeating screening of LTBI within the same population may present some practical issues that are not considered in this modelling study. It can be difficult to interpret positive tests in previously treated individuals, since LTBI tests frequently remain positive after treatment. Thus, some individuals may be treated unnecessarily if all positive tests were interpreted as evidence of current infection. Conversely, reinfection occurs, and some infections may be missed if positive tests were systematically interpreted as false-positives in previously treated individuals, in which case testing would become redundant for this population. These issues suggest that repeating active screening programmes every 5 years or 10 years may be more realistic than 2-year cycles. They also highlight the importance of developing highly accurate infection screening tools that could distinguish between previous and current infections or identify biomarkers that could predict short-term progression risk.

## Conclusion

In conclusion, our analysis suggests that the Marshall Islands may not reach all the End TB Strategy targets under the current programmatic situation. However, it would be possible to achieve the 2035 End TB targets through periodic repetition of the same interventions as those already implemented in the country, using a similar screening rate at the national level. If this were to occur, it would be a rare example of the capability of existing tools to achieve End TB targets.

## Ethics approval

Ethics approval was not needed for this study as it did not involve human subjects.

## Data availability

The data underlying this article and the code used to perform the analyses are available in a Github repository at [https://github.com/monash-emu/AuTuMN/releases/tag/TB_Marshall_Islands_Int_J_Epidemiol].

## Supplementary data

Supplementary data are available at *IJE* online.

## Author contributions

R.R., B.M.W., T.I. and J.M.T. designed the study. A.L., J.N.J., T.J. and M.K.L. contributed to collecting and processing the data used to inform the model. R.R., B.M.W., E.S.M. and J.M.T. designed the mathematical model. R.R. and J.M.T. implemented the code. R.R. conducted the experiments. All authors contributed to the interpretation of the findings. R.R. wrote the first version of the manuscript. All authors contributed to the final version of the manuscript.

## Funding

This work was funded by the Australian National Health and Medical Research Council (Project Grant APP1144570 and Fellowship Grant APP1142638) and the Western Pacific Regional Office of the World Health Organization.

## Supplementary Material

dyac045_Supplementary_Data
