# Supplementary material for: Estimating the long-term effects of mass screening for latent and active tuberculosis in the Marshall Islands
Source: Int J Epidemiol. 2022 Mar 22;51(5):1433–45. doi: 10.1093/ije/dyac045 (PMC9557838; doi:10.1093/ije/dyac045)
Supplement: dyac045_Supplementary_Data [file dyac045_supplementary_data.pdf]

Estimating the long-term effects of mass screening for latent and active tuberculosis in the Marshall Islands

Romain Ragonnet, Bridget M Williams, Angela Largen, Joaquin Nasa Jr, Tom Jack, Mailynn K Langinlur, Eunyoung Ko, Kalpeshsinh Rahevar, Tauhid Islam, Justin Denholm, Ben J Marais, Guy B Marks, Emma S McBryde, James M Trauer

Supplementary Material

Contents

Contents 1
Model design 2
  Base model 2
  Stratification by organ status 2
  Stratification by age 2
  Stratification by location 3
  Births and deaths 4
  M.tb transmission 4
Progression parameters 5
  Progression from latent to active TB 5
  Effect of diabetes 5
  Natural history flows 5
  Passive detection of active TB 5
  Treatment outcomes 6
Modelled interventions 6
  Population-based screening and treatment of LTBI 6
  Active case finding 6
  Calculation of the screening rates 7
Model calibration 7
  Overall approach 7
  Parameters' prior distributions 7
  Calibration targets 7
  Posterior estimates of calibrated parameters 9
Historical TB epidemic trajectory 9
Sensitivity analyses 10
  Considering various levels of future diabetes prevalence 10
  Considering alternate values for the sensitivity of LTBI screening 11
  Using a different assumption for the effect of BCG vaccination 11

Assuming that passive case detection has been constant since 1980 13
Assuming more inter-island mixing 16
Benefit risk of active screening interventions 19
Projected impact of periodic interventions (log scale) 19
References 20

Model design

Base model

We use a deterministic compartmental model including six types of compartments that represent different states of infection and disease. The model uses the same conceptual approach and similar assumptions to previously published models (1–4). Here we describe the model structure before applying any stratification. A susceptible compartment (*S*) is used to represent individuals who have never been infected with *Mycobacterium tuberculosis* (M.tb). Latent TB infection (LTBI) is modelled using two successive compartments: early latent (*E*) and late latent (*L*) to capture the declining risk of disease progression over time from infection (5). The active disease compartment (*I*) represents individuals who have progressed to the active stage of TB disease. Diseased individuals who recover through self-cure progress directly to the recovered compartment (*R*). All diseased individuals who are detected are assumed to be started on treatment (compartment *T*). Treatment may result in cure (progression to *R*), relapse (return to *I*) or death.

Non-TB-related mortality is modelled by applying death rates to all model compartments. In addition, disease-specific mortality is implemented by applying increased mortality rates to the active disease compartments (*I* and *T*).

Reinfection occurs in the model in two different ways. First, latently infected individuals may be reinfected, with this process modelled using a flow from the late latent (*L*) to the early latent compartment (*E*). Second, individuals who have recovered from TB disease may be reinfected and return to the early latent compartment. The structure of our model allows for differential risk of infection for the currently and previously infected individuals, compared to the infection-naive individuals.

Figure 1 (main text) represents the model structure.

Stratification by organ status

The model explicitly includes three types of TB clinical manifestations, based on the organ affected by the disease and the smear status. The three "organ categories" are smear-positive TB, smear-negative pulmonary TB and extrapulmonary TB. The term "smear-negative TB" will be used to refer to "smear-negative pulmonary TB" hereafter.

The stratification by organ status applies to the active disease compartments (*I* and *T*), and we assume differential disease fatality rates, infectiousness levels and detection rates by organ status.

Stratification by age

The model is stratified using five categories: 0-4, 5-14, 15-34, 35-49 and 50+ years old. We assume heterogeneous mixing by age using an age-specific contact rate matrix. Since no local estimates of contact patterns by age were available for the Marshall Islands, we used a contact survey conducted in the Fijian population and adjusted the estimates to account for age distribution differences between the two countries (6). The modelled average daily number of contacts that an individual aged i has with individuals aged j (denoted c_{ij}) is calculated as:

$$c_{ij} = q_{ij} \times \frac{\pi_j}{\kappa_j},$$

where q_{ij} is the contact rate (homologous to c_{ij}) obtained from the Fijian survey, π_j is the proportion of the Fijian population aged j and κ_j is the proportion of the Marshall Islands population aged j.

The original Fijian matrix was calculated using the following age groups: 0-4, 5-14, 15-34, 35-54 and 55+ years old. The adjusted matrix c_{ij} was then converted to match the model's age groups by aggregating the contact rates across the relevant age groups and applying weighted averages based on the population age distribution. The code used to generate the contact matrix is available on Github (https://github.com/monash-emu/AuTuMN/blob/master/autumn/tools/inputs/social_mixing/build_synthetic_matrices.py).

Stratification by location

All model compartments are further stratified by location to explicitly represent the respective populations of the Majuro Atoll, the Ebeye Island and other islands. Population proportions by location were informed by the 2011 National Census. We assumed that individuals make 95% (80% in sensitivity analysis) of their contacts with persons living in the same geographic stratum. The remaining 5% (20% in sensitivity analysis) are distributed between the two other locations and the relative contribution of each of the other two regions calculated based on its population size. The three locations shared the same values for most model parameters, but we allowed for differential passive screening rates by location, and the interventions were only implemented in the Ebeye Atoll (ACF) and in the Majuro Atoll (ACF and LTBI screening).

Table S1 summarises the different stratifications implemented in the model and highlights the key changes applied to the stratified parameters.

Stratification	Strata	Significance to model
Age	0-4 years old<br>5-14 years old<br>15-34 years old<br>35-49 years old<br>50 years and over	• Risk of progression from latent to active TB varies with age.<br>• Background mortality rates vary with age.<br>• Age-specific infectiousness.<br>• Diabetes prevalence varies with age.<br>• Heterogenous mixing by age<br>• BCG vaccine effect and coverage varies with age.
Location	Majuro<br>Ebeye<br>Other	• Heterogenous mixing between the three geographical groups will simulate the impact of inter-island travel on the population effect of the interventions in Ebeye and Majuro. 95% (80% in sensitivity analysis) of social contacts occur with individuals from the same region. The remaining proportion of contacts was split between the other two regions, weighting for their respective population.<br>• Case detection rates may vary between the regions<br>• The rates of LTBI screening and active case finding vary by location
Organ status	Pulmonary smear-positive<br>Pulmonary smear-negative<br>Extrapulmonary	• Case detection rates vary according to organ status.<br>• Smear-positive TB has a higher level of infectiousness than smear-negative TB, and extrapulmonary TB is considered non-infectious.

Table S1. Summary of model stratifications

Births and deaths

Births are modelled using time-variant crude birth rates that are multiplied by the modelled population size to determine the number of newborn individuals entering the model at each time. A time-variant and age-specific rate or non-TB-related mortality applies to all model compartments to simulate deaths from other causes than TB. We use estimates from the UN population division to inform the birth and mortality rates. No data specific to the Marshall Islands were available, so we used the crude birth rates and mortality rates by age of the Federated States of Micronesia.

We also apply additional death rates to the compartments I and T to reflect mortality induced by TB disease.

M.tb transmission

We use different levels of susceptibility to infection for individuals who are currently latently infected with M.tb or have recovered from active TB, as compared to infection-naive individuals. The effect of BCG vaccination is captured by reducing the susceptibility to infection of individuals under the age of 30 years old. We assume a 70% reduction in the susceptibility of BCG-vaccinated children under the age of 15 years old (7). A linear function is used to reflect the progressive loss of BCG immunity between the age of 15 and 30 years old. Figure S1 presents the profile of BCG immunity wane. The continuous wane profile is then automatically converted to a step function such that each model's age band is associated with the average value of the wane function over the relevant interval. The susceptibility adjustment induced by BCG is also based on the country's time-variant BCG coverage as reported by the WHO. The rest of this section describes the different infectiousness adjustments implemented in the model.

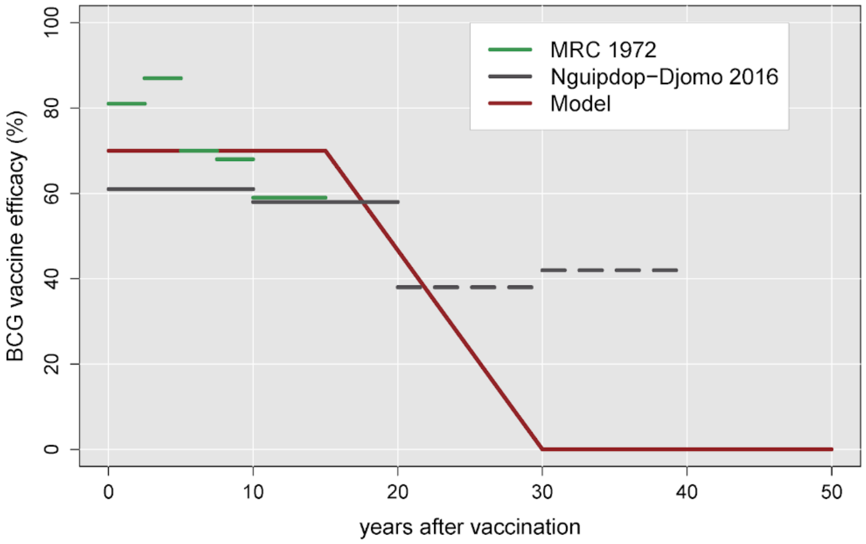

Figure S1. Assumed wane profile of BCG efficacy.
Green and grey lines represent estimates obtained from literature (7,8), while the red line shows the modelled vaccine effect. Dashed lines show estimates associated with non-significant efficacy.

We assume that smear-negative TB is 25% as infectious as smear-positive TB, while extrapulmonary TB is modelled as a non-infectious disease state (9,10).

Infectiousness is assumed higher for older individuals, and we use the sigmoidal function $age \rightarrow \frac{1}{1+e^{-(age-15)}}$ to model a progressive increase with age (11). The continuous age profile of

infectiousness is then automatically converted to a step function, similarly to what was previously described for the immunity wane profile of BCG vaccination.

Finally, individuals who are on treatment are assumed to be partially infectious, as infectiousness declines rapidly after treatment initiation. Infectiousness is multiplied by 0.08 for the treatment compartment compared to the untreated disease compartment to reflect the fact that individuals may remain infectious for two weeks out of the 26 weeks of a standard regimen.

Progression parameters

Progression from latent to active TB

We use the estimates reported in Ragonnet *et al.* to inform the modelled dynamics of activation from latent to active TB (Table 2, main text) (5). These parameters vary by age, and a multiplier is used to incorporate uncertainty around the progression rates (5).

Effect of diabetes

The model is not stratified by diabetes status. Instead, we model the effect of diabetes type 2 by increasing the rates of progression from latent to active TB using age-specific multipliers. For each age group, the value of the diabetes-effect multiplier depends on the age-specific proportion of diabetic individuals and the relative rate of TB reactivation for diabetic individuals compared to non-diabetic individuals (see Table 2, main text). The multiplier applied to the TB progression rates can be written $m_i = d_i \times rr_{diabetes} + 1 - d_i$, where d_i is the proportion of diabetic individuals in the age-group i and $rr_{diabetes}$ is the relative rate of TB reactivation for diabetic individuals compared to non-diabetic individuals.

Diabetes prevalence was assumed to increase progressively to reach the values presented in Table 2 in 2020 (main text) and was then assumed to be constant after that time in the base case analysis. However, we considered both a linear increase of 20% and a linear decrease of 20% by 2050 in sensitivity analyses.

Natural history flows

We use the estimates reported in Ragonnet *et al.* to model the rate of TB mortality in the absence of treatment and the rate of self-recovery (12). We use different rates of untreated TB mortality and self-recovery for smear-positive TB compared to smear-negative TB. The TB mortality and self-recovery rates associated with extrapulmonary TB are assumed to be the same as those of smear-negative TB.

Passive detection of active TB

The detection rate is defined as the rate of progression from the active disease to the treatment compartment, as all detected individuals are assumed to be started on treatment at diagnosis in our model. This rate is calculated by multiplying the screening rate with the diagnostic test sensitivity. The screening rate can be interpreted as the reciprocal of the average time that diseased individuals take to seek care. The diagnostic sensitivity varies according to the organ status to reflect the relative differences in the difficulty to diagnose smear-negative TB and extrapulmonary TB, as compared to smear-positive TB.

We use a time-variant function to model the screening rate in order to capture detection improvements over time. This process is modelled assuming a continuous increase in the screening rate through the following function:

$t \rightarrow 0.5(\tanh(m(t-c)+1) \times final$, where c is the time at which the curve inflects, m is the value of the gradient at the inflection point (shape parameter), and $final$ is the upper asymptote value. The parameters c, $final$ and m are varied during calibration, and Figure S2 presents the time-variant profile of screening rate obtained by sampling these parameters from their posterior joint distribution.

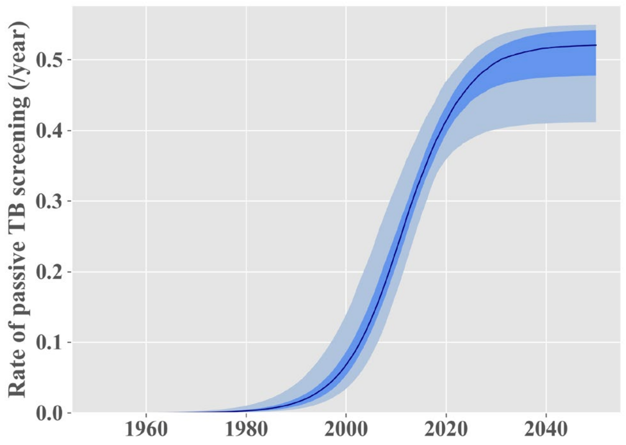

Figure S2. Posterior estimate of the passive screening rate profile. The solid lines represent the median estimates. The shaded areas show the interquartile ranges (dark shade) and 95% credible intervals (light shade).

Treatment outcomes

Treated individuals can experience three different treatment outcomes: treatment success, relapse or death. The rate of treatment-induced recovery (φ) is set to the reciprocal of the duration of a completed treatment course. We then use the observed treatment success proportion (often referred to as "treatment success rate") as model input. In our model, it is calculated from $TSR = \frac{\varphi}{\varphi+\rho+\mu_T+\mu}$, where ρ is the relapse rate, μ_T is the excess mortality rate of individuals on TB treatment, and μ is the non-TB-related mortality rate. Finally, we calculate the respective values of ρ and μ_T using the observed proportion of deaths among all negative treatment outcomes, denoted π. We have $\pi = \frac{\mu_T+\mu}{\rho+\mu_T+\mu}$ that we inject into the TSR equation. After calculations, we obtain:

$\mu_T = \pi\varphi\frac{1-TSR}{TSR} - \mu$ and $\rho = (\mu_T + \mu)(\frac{1}{\pi} - 1)$.

Note that we need to verify $\varphi \geq \frac{\mu}{\pi} \times \frac{TSR}{1-TSR}$ to ensure that $\mu_T \geq 0$. If this condition is not verified, which may be the case if both the treatment success rate and the rate of non-TB-related mortality are high, we force $\varphi = \frac{\mu}{\pi} \times \frac{TSR}{1-TSR}$.

Since the non-TB-related mortality rate varies by age, the values of the treatment-induced recovery (φ), relapse rate (ρ) and mortality rate of individuals on TB treatment (μ_T) also vary by age.

Modelled interventions

Population-based screening and treatment of LTBI

Mass LTBI screening and treatment is implemented as part of the intervention conducted in Majuro in 2018. This is modelled by making latently infected individuals (from *E* and *L*) transition to the recovered compartment (*R*). The rate associated with these flows is obtained by multiplying the LTBI screening rate with the sensitivity of the LTBI test employed and the individual-level efficacy of preventive treatment. The LTBI screening rate is implemented as a time-variant parameter that is stratified by location.

Active case finding

Active case finding (ACF) is implemented to simulate the interventions linked to the detection of individuals with active TB implemented in Ebeye in 2017 and in Majuro in 2018. This is modelled by implementing an additional transition flow from compartment *I* to compartment *T*. The rate associated with this flow is obtained by multiplying the location-specific ACF screening rate with the sensitivity of the detection algorithm used for the ACF intervention. The ACF screening rate is implemented as a time-variant parameter.

Calculation of the screening rates

To simulate the interventions, we apply a positive rate of ACF and/or LTBI screening over the intervention periods. The screening rates are determined such that the modelled total proportion of the population screened corresponds to the true population proportion screened. The screening rate is set equal to $-\log(1-coverage)$ for the year during which the intervention is implemented, where $coverage$ is the total proportion of the population screened by the intervention. In the Ebeye Atoll, it was estimated that 85% of adult individuals (aged 15 years old and over) were screened for active TB. In the Majuro Atoll, 22,623 individuals out of a population of 27,797 (81%) were screened for both LTBI and active TB ($coverage = 81\%$). However, this proportion was then reduced to to account for incomplete screening completion (86.5% with TST reading) and for the fact that some individuals diagnosed with LTBI were not started on treatment (85% either not recommended for treatment or refused treatment). As a result, effective LTBI screening coverage among the latently infected population then fell to 59%.

The screening rates obtained from the Majuro intervention was used to model the intervention scenarios involving country-wide screening repeated periodically.

Model calibration

Overall approach

The model is calibrated using an adaptive Metropolis (AM) algorithm. We use the multidimensional Gaussian distribution with variable covariance matrix presented by Haario and colleagues to sample parameters from their posterior distributions (13). We ran seven independent AM chains to sample the calibrated parameters. We discarded the first 2000 iterations of each chain as burn-in and the algorithm was run such that more than 15,000 post-burn-in iterations were obtained for each chain. We then combined the samples of the seven chains to project epidemic trajectories over time.

The likelihood function was derived from comparing model outputs to target data at each time point nominated for calibration. We used normal distributions centred on the model predictions, and the standard deviations of these distributions were manually calibrated to optimise sampling efficiency.

Parameters' prior distributions

We use uniform prior distributions characterised by the intervals presented in Table 2 (main text) and Table S2 below.

Calibration targets

The following table summarises the calibration targets used to calculate the likelihood of the AM algorithm.

Parameter	Range
Initial population size	200 - 800
Transmission scaling factor	0.002 – 0.01
Progression multiplier	0.5 - 2.0
Screening profile (inflection time), year	2000.0 - 2020.0
Screening profile (shape)	0.07 - 0.1
Screening profile (final rate), per year	0.4 - 0.55
Relative rate of passive TB screening in Ebeye (ref. Majuro)	1.3 - 2.0
Relative rate of passive TB screening in other islands (ref. Majuro)	0.5 - 1.5
Relative rate of TB progression for diabetic individuals	2.0 - 5.0
Relative risk of infection for individuals with latent infection (ref. Infection-naive)	0.2 - 0.5
Relative risk of infection for individuals with history of infection (ref. Infection-naive)	0.2 - 1.0
Efficacy of preventive treatment	0.75 - 0.85
Relative screening rate following ACF interventions (ref. Before intervention)	1.0 - 1.5
TB mortality (smear-positive), per year	0.335 - 0.449
TB mortality (smear-negative), per year	0.017 - 0.035
Self-cure rate (smear-positive), per year	0.177 - 0.288
Self-cure rate (smear-negative), per year	0.073 - 0.209

Table S2. Prior distribution ranges

Variable	Targeted value	Source
TB prevalence in Majuro in 2018	1366 per 100,000 persons	Measured during intervention
TB prevalence in Ebeye in 2017	755 per 100,000 persons	Measured during intervention
LTBI prevalence in Majuro in 2018	38%	Measured during intervention, adjusted for test sensitivity.
TB notifications in Majuro in<br>• 2012<br>• 2013<br>• 2014<br>• 2015<br>• 2016<br>• 2017<br>• 2018<br>• 2019<br>• 2020	 • 91<br>• 116<br>• 115<br>• 90<br>• 119<br>• 116<br>• 376<br>• 135<br>• 96	TB program
TB notifications in Ebeye in<br>• 2012<br>• 2013<br>• 2014<br>• 2015<br>• 2016<br>• 2017<br>• 2018<br>• 2019<br>• 2020	 • 44<br>• 27<br>• 30<br>• 29<br>• 53<br>• 80<br>• 30<br>• 45<br>• 39	TB program
Total population size in 2011	53158	2011 National Census

Table S3. Calibration targets

Posterior estimates of calibrated parameters

Table S4 presents the posterior estimates obtained for the fitted parameters. The percentiles were obtained after combining the samples from the seven AM chains.

Parameter	2.5th percentile	Median	97.5th percentile
Initial population size	333	414	519
Transmission scaling factor	0.0038	0.0043	0.0057
Progression multiplier	1.52	1.83	1.9
Screening profile (inflection time), year	2010	2010	2010
Screening profile (shape)	0.0709	0.0854	0.0994
Screening profile (final rate), per year	0.414	0.522	0.55
Relative rate of passive TB screening in Ebeye (ref. Majuro)	1.44	1.79	2
Relative rate of passive TB screening in other islands (ref. Majuro)	0.534	1.02	1.48
Relative rate of TB progression for diabetic individuals	3.99	4.98	4.99
Relative risk of infection for individuals with latent infection (ref. infection-naive)	0.209	0.315	0.495
Relative risk of infection for individuals with history of infection (ref. infection-naive)	0.221	0.501	0.97
Efficacy of preventive treatment	0.754	0.808	0.849
Relative screening rate following ACF interventions (ref. before intervention)	1.01	1.26	1.49
TB mortality (smear-positive), per year	0.335	0.339	0.432
TB mortality (smear-negative), per year	0.0171	0.0235	0.034
Self-cure rate (smear-positive), per year	0.177	0.192	0.286
Self-cure rate (smear-negative), per year	0.073	0.073	0.0731

Table S4. Parameter posterior estimates

Historical TB epidemic trajectory

The model was initialised by seeding one infectious individual in 1800 in a fully-susceptible population. This approach allowed M.tb transmission to emerge naturally over the last two centuries until reaching the calibration targets in recent years. Figure S3 represents the historical epidemic trajectory.

The model captured substantial uncertainty during the first 200 simulated years due to the absence of historical data for that period, but uncertainty is then refined for the most recent years. We simulated a progressive incidence increase until around 1900, followed by a nearly stable phase until around 1950. The estimated TB incidence then increased again significantly due to the emergence of diabetes. The increasing incidence trend was estimated to peak and decline around 1990 as TB detection and BCG coverage increased.

The TB mortality and LTBI prevalence trajectories followed that of incidence, whereas the modelled TB notifications only emerged after around 1970, which is a consequence of the modelled profile of case detection (see Figure S2).

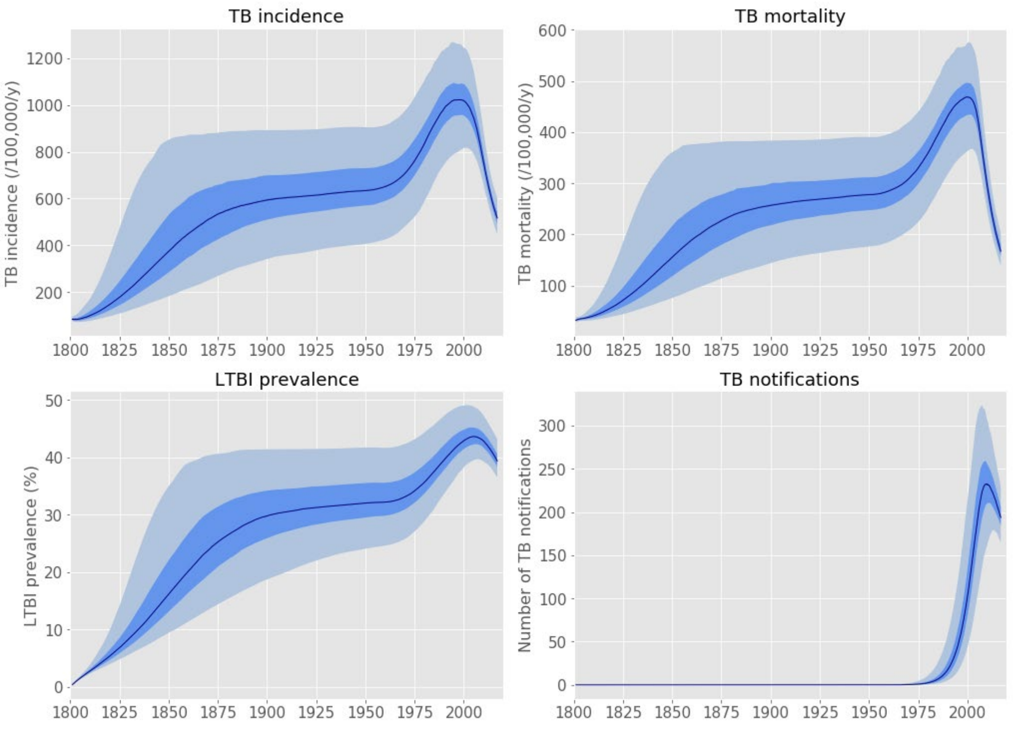

Figure S3. Historical epidemic trajectory projected by the model. The solid lines represent the median estimates. The shaded areas show the interquartile ranges (dark shade) and 95% credible intervals (light shade).

Sensitivity analyses

Considering various levels of future diabetes prevalence

The results of our analysis considering different assumptions for the future trend of diabetes prevalence are shown in Figure S4. This analysis included the interventions previously conducted in Majuro and Ebeye and assumed that TB control would be similar to the current programmatic situation until 2050. We considered both a linear increase of 20% (red line and shade) and a linear decrease of 20% (green line and shade) in diabetes prevalence between 2020 and 2050 in this sensitivity analysis.

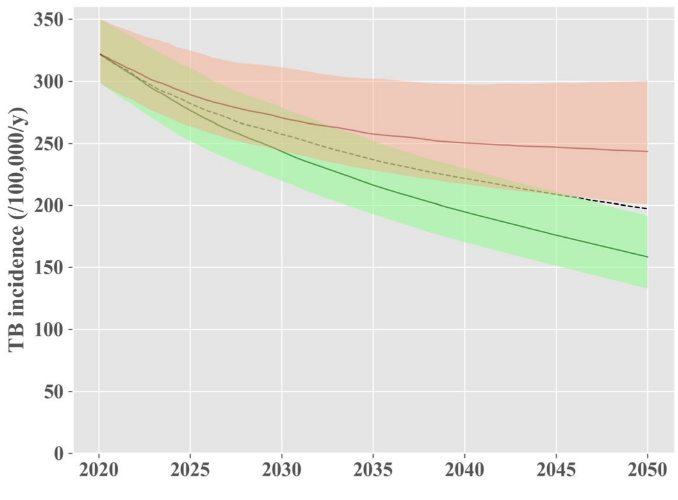

Figure S4. Projected epidemic under status-quo scenario considering different future trends for diabetes prevalence. The dashed line represents the base case median estimate associated with a constant future diabetes prevalence, maintained at the same level as in 2020. The green (resp. red) line shows the median projection associated with a 20% decrease (resp. increase) in diabetes prevalence by 2050. The shades represent the interquartile ranges.

Considering alternate values for the sensitivity of LTBI screening

We assumed a sensitivity of 75% for LTBI screening in our base-case analysis. In a sensitivity analysis, we conducted simulations considering LTBI screening sensitivity values ranging from 50% to 90%. Figure S5 shows the main epidemic indicators under the status-quo scenario including the 2017-2018 interventions, without repeated screening in the future.

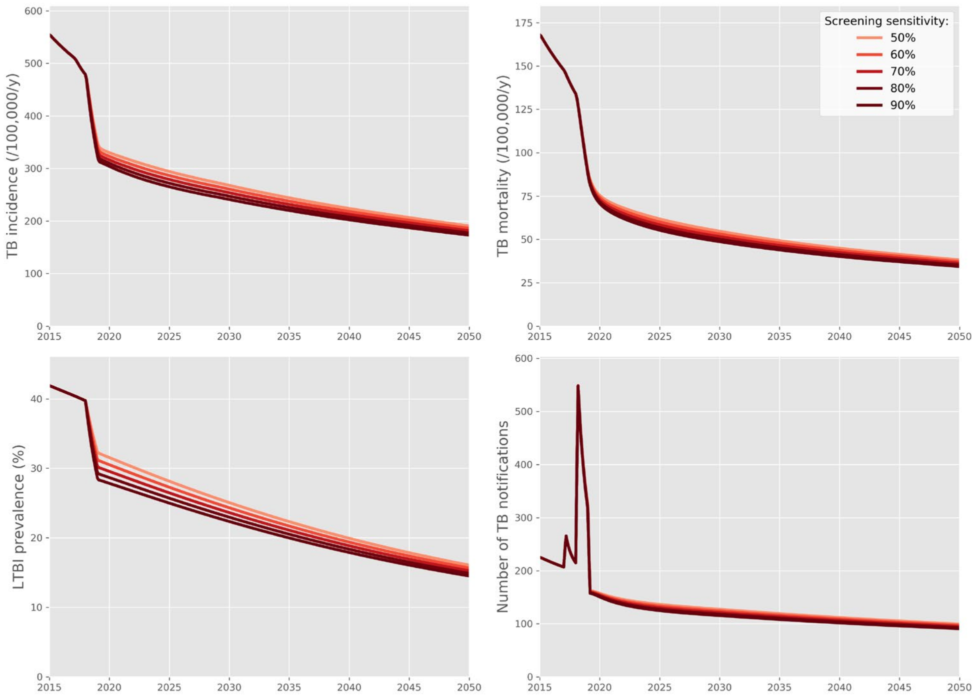

Figure S5. Projected epidemic under status-quo scenario considering different values of LTBI screening sensitivity. This analysis considered the "status-quo" scenario including the 2017-2018 interventions. We used the maximum likelihood estimates obtained during calibration (i.e. the best fitted run) to inform the parameters used in this analysis.

Using a different assumption for the effect of BCG vaccination

There remains uncertainty around the nature of the protective effect of BCG vaccination. In the base-case analysis, BCG was assumed to protect against M.tb infection such that the transmission rate governing the transition between the *S* compartment and the *E* compartment was affected by BCG. In a sensitivity analysis, we assumed that BCG does not prevent infection, but only reduces the risk of mortality in individuals with active TB. We used the same approach as in the base-case analysis to characterise BCG efficacy by age and BCG coverage over time (see "M.tb transmission" section).

Figures S6 and S7 present the same outputs as in the main text regarding the effect of the 2017-2018 interventions and that of periodic interventions repeated in the future, respectively, this time considering the alternate assumption for BCG effect.

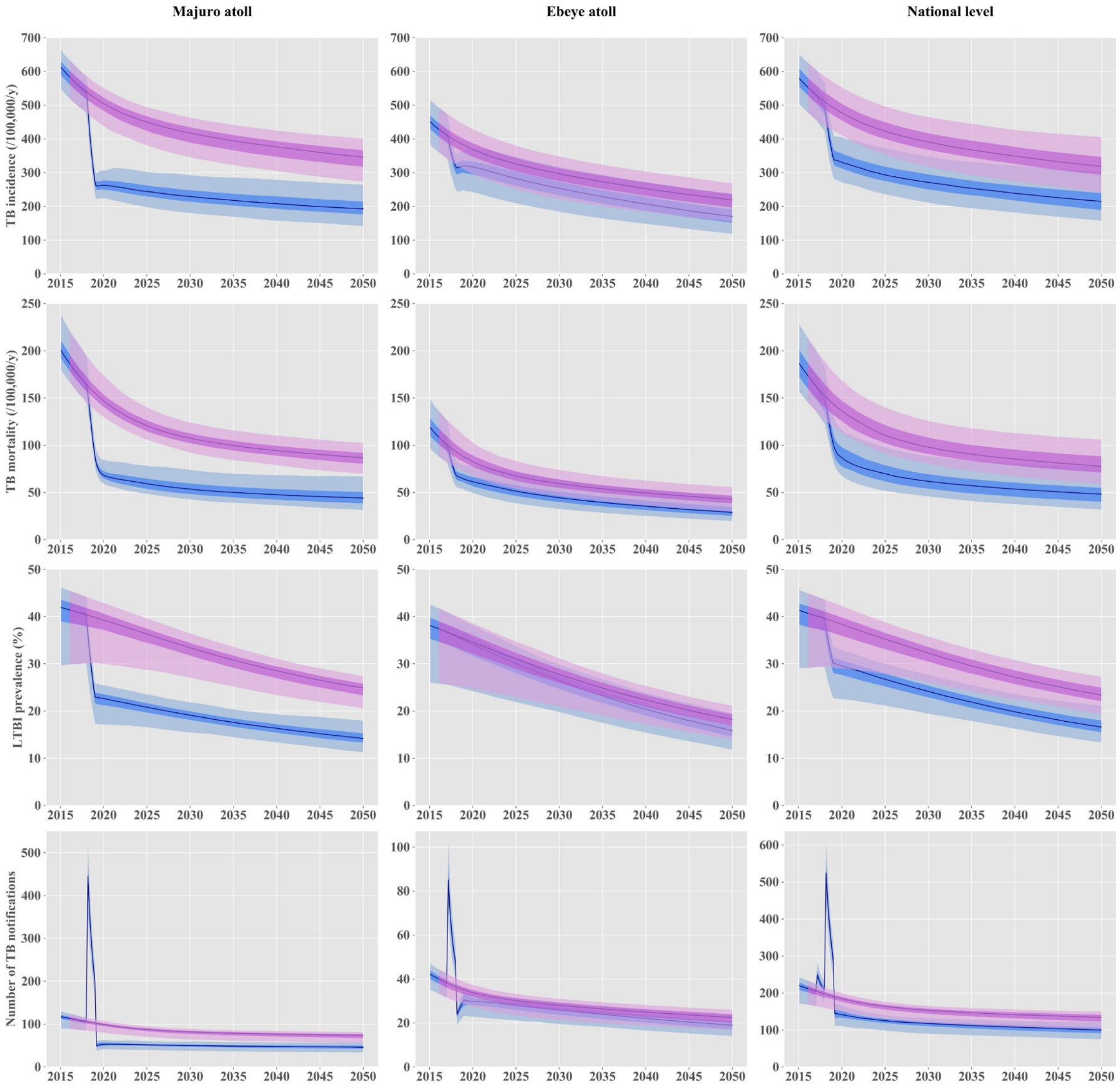

Figure S6. Projected effect of the active screening interventions implemented in 2017 and 2018, assuming BCG only reduces the risk of death from TB.

The solid lines represent the median estimates. The shaded areas show the interquartile ranges (dark shade) and 95% credible intervals (light shade) projected in the absence of any intervention (pink) and under a scenario including the interventions implemented in 2017-2018 in Majuro and Ebeye (blue).

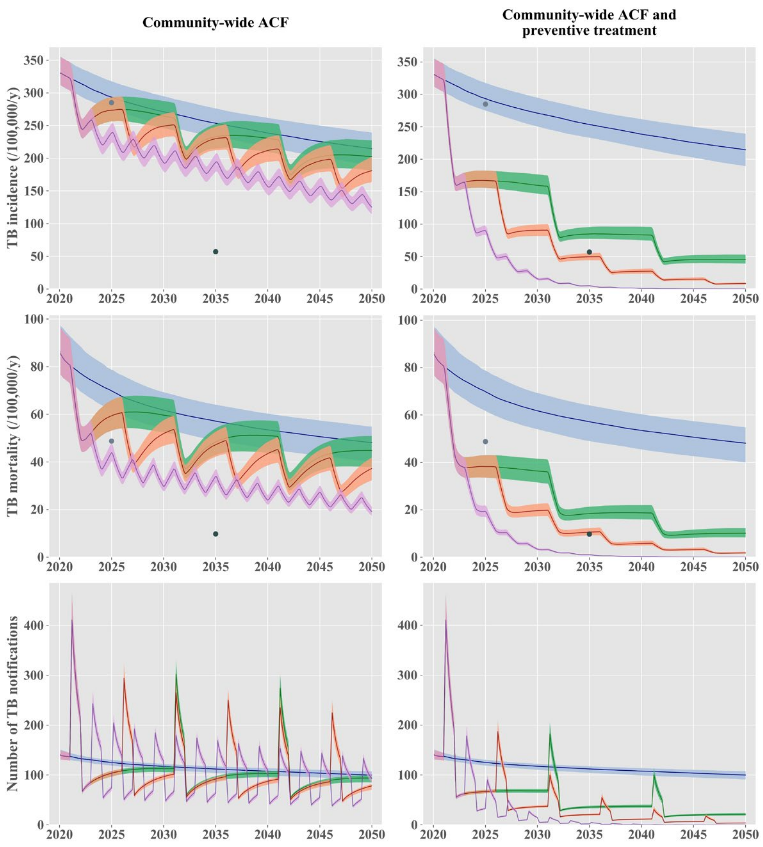

Figure S7. Projected effect of periodic community-wide interventions, assuming BCG only reduces the risk of death from TB.
The solid lines represent the median estimates, and the shaded areas show the interquartile credible ranges. The "status-quo" scenario is represented in blue in all panels. The left column of panels presents scenarios including nationwide active case finding (ACF) repeated every two years (purple) or every five years (orange) or every ten years (green). The right column of panels presents nationwide ACF scenarios combined with mass latent infection screening and treatment, repeated every two years (purple) or five years (red). The light and dark grey dots show the 2025 milestones and the 2035 targets, respectively, according to the End TB Strategy.

Assuming that passive case detection has been constant since 1980

Although TB case detection has undoubtedly increased over time due to improvements in TB control, the profile of this scale-up remains uncertain. Including available data on active TB prevalence (in 2017-2018) and TB notifications in our model calibration process allowed estimation of the rate of passive screening for the most recent years. However, in the absence of any TB prevalence data before 2017 we had to make assumptions regarding the historical scale-up profile of passive screening. In particular, we allowed the inflection time (i.e. the time when improvements in passive screening were the fastest) to vary between 2000 and 2020 in the base-case analysis, based on discussions with the country TB program. Such recent case detection improvements were partly responsible for the projected decline in the epidemic trajectory, even under the counterfactual scenario where active screening interventions were excluded.

In a sensitivity analysis, we considered an alternate assumption regarding the passive case detection profile. We assumed that improvements in passive screening occurred before 1980 and that the passive screening rate remained constant thereafter. This was done by reusing the same approach presented in the Passive detection of active TB section, while fixing the value of the shape parameter ($m = 0.2$) and the inflection time ($c = 1960$), whereas the final value of passive screening rate ($final$) remained automatically calibrated. Figure S8 represents the posterior estimate of the passive screening profile obtained in this sensitivity analysis.

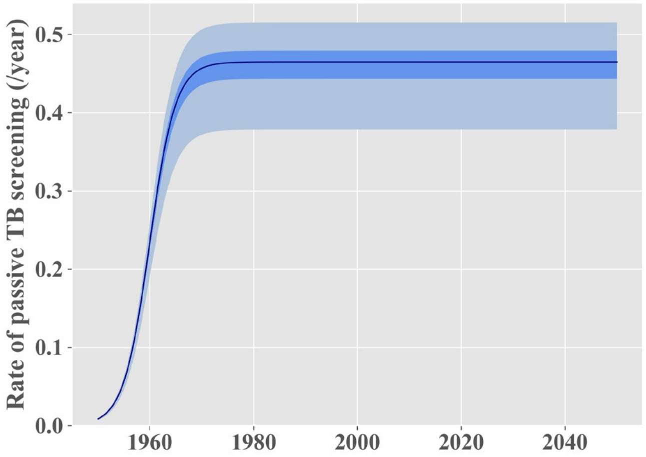

Figure S8. Posterior estimate of the passive screening rate profile under the alternate assumption regarding detection scale-up over time. The solid lines represent the median estimates. The shaded areas show the interquartile ranges (dark shade) and 95% credible intervals (light shade).

Figures S9 and S10 present the same outputs as in the main text regarding the effect of the 2017-2018 interventions and that of periodic interventions repeated in the future, respectively, this time considering the alternate passive screening profile.

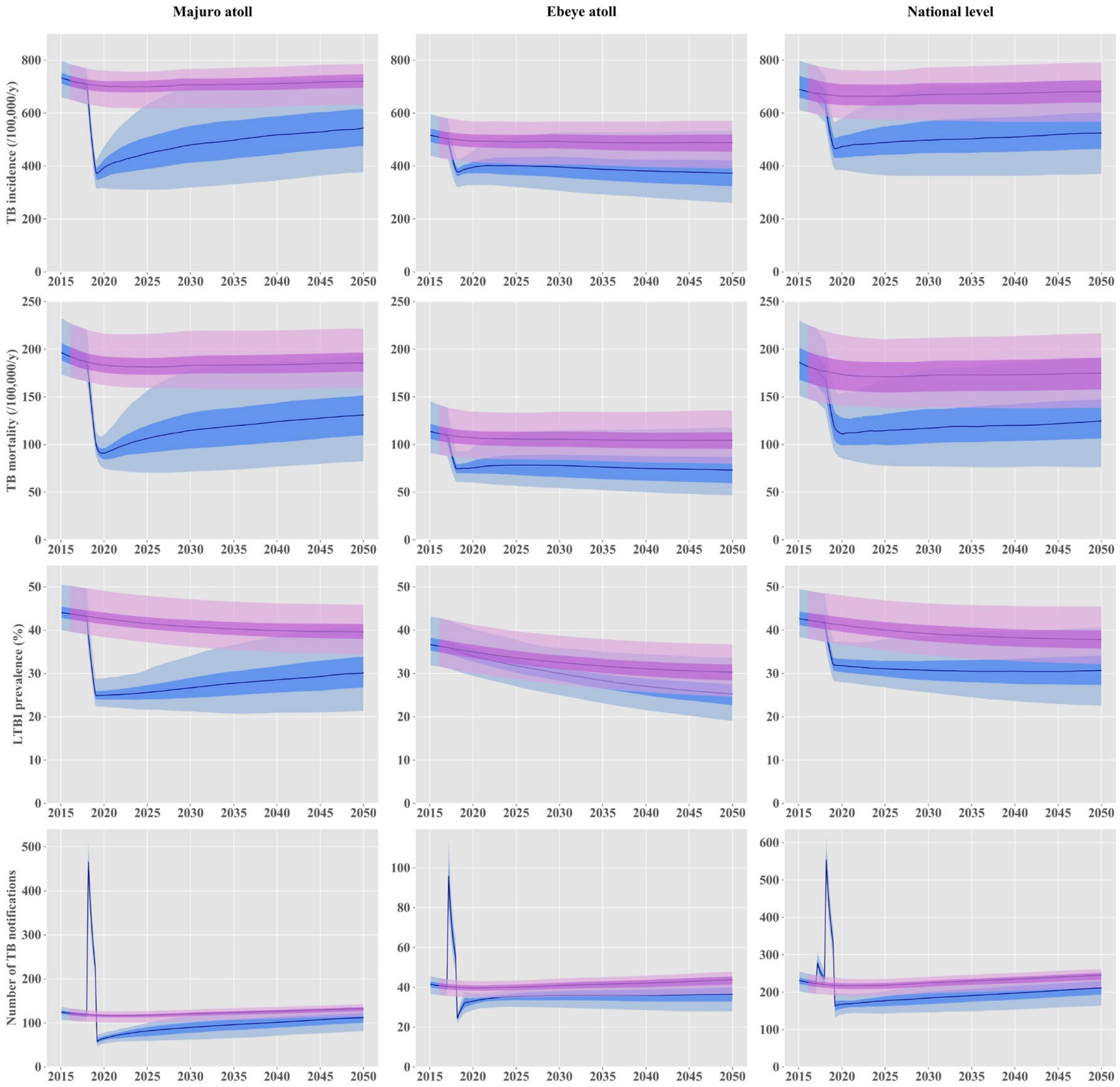

Figure S9. Projected effect of the active screening interventions implemented in 2017 and 2018, assuming an alternate profile of passive case detection over time.

The solid lines represent the median estimates. The shaded areas show the interquartile ranges (dark shade) and 95% credible intervals (light shade) projected in the absence of any intervention (pink) and under a scenario including the interventions implemented in 2017-2018 in Majuro and Ebeye (blue).

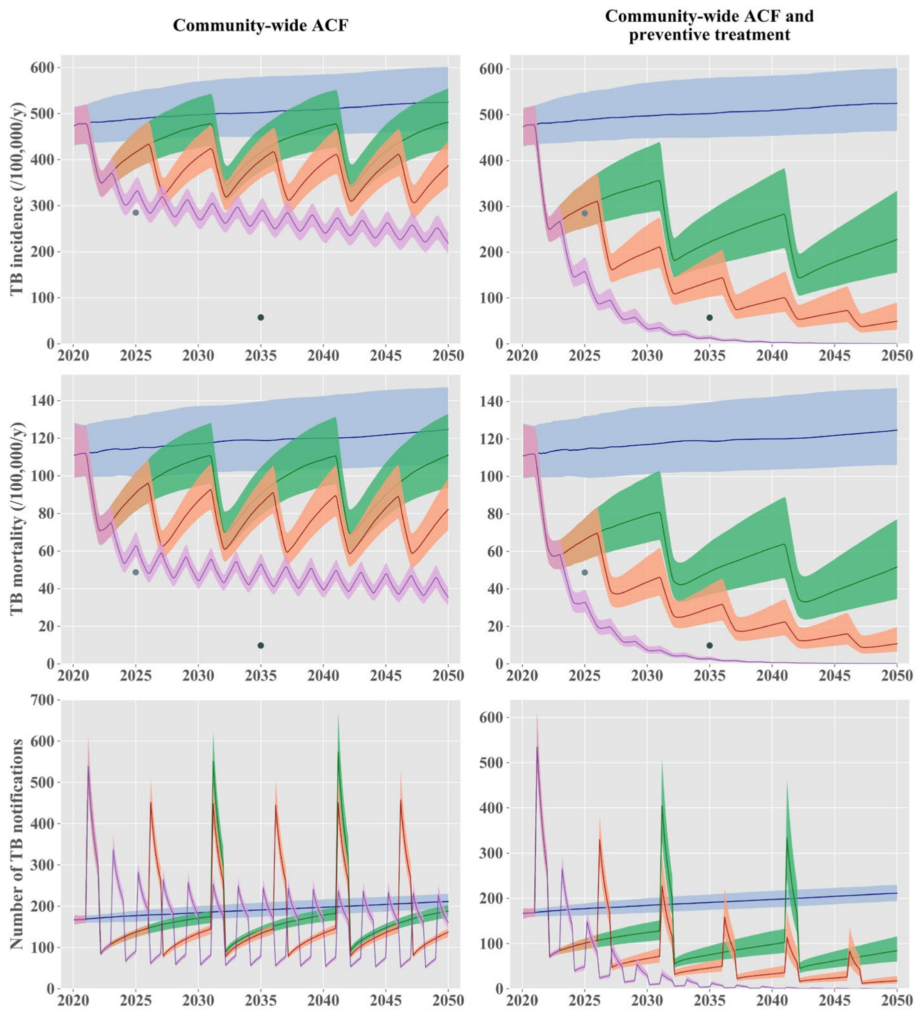

Figure S10. Projected effect of periodic community-wide interventions, assuming an alternate profile of passive case detection over time.
The solid lines represent the median estimates, and the shaded areas show the interquartile credible ranges. The "status-quo" scenario is represented in blue in all panels. The left column of panels presents scenarios including nationwide active case finding (ACF) repeated every two years (purple) or every five years (orange) or every ten years (green). The right column of panels presents nationwide ACF scenarios combined with mass latent infection screening and treatment, repeated every two years (purple) or five years (red). The light and dark grey dots show the 2025 milestones and the 2035 targets, respectively, according to the End TB Strategy.

Assuming more inter-island mixing

In the base-case analysis, we assumed that a typical Marshall Islands inhabitant has 95% of his/her inter-personal contacts with individuals living on his/her own island. The remaining 5% would be contacts with individuals living on other islands. In a sensitivity analysis, we modified this assumption to consider 80% of contacts occurring within the same island group and 20% with individuals from other island groups, with the same assumption concerning the distribution of these contacts across the other two islands.

Figures S11 and S12 present the results assuming increased inter-island mixing.

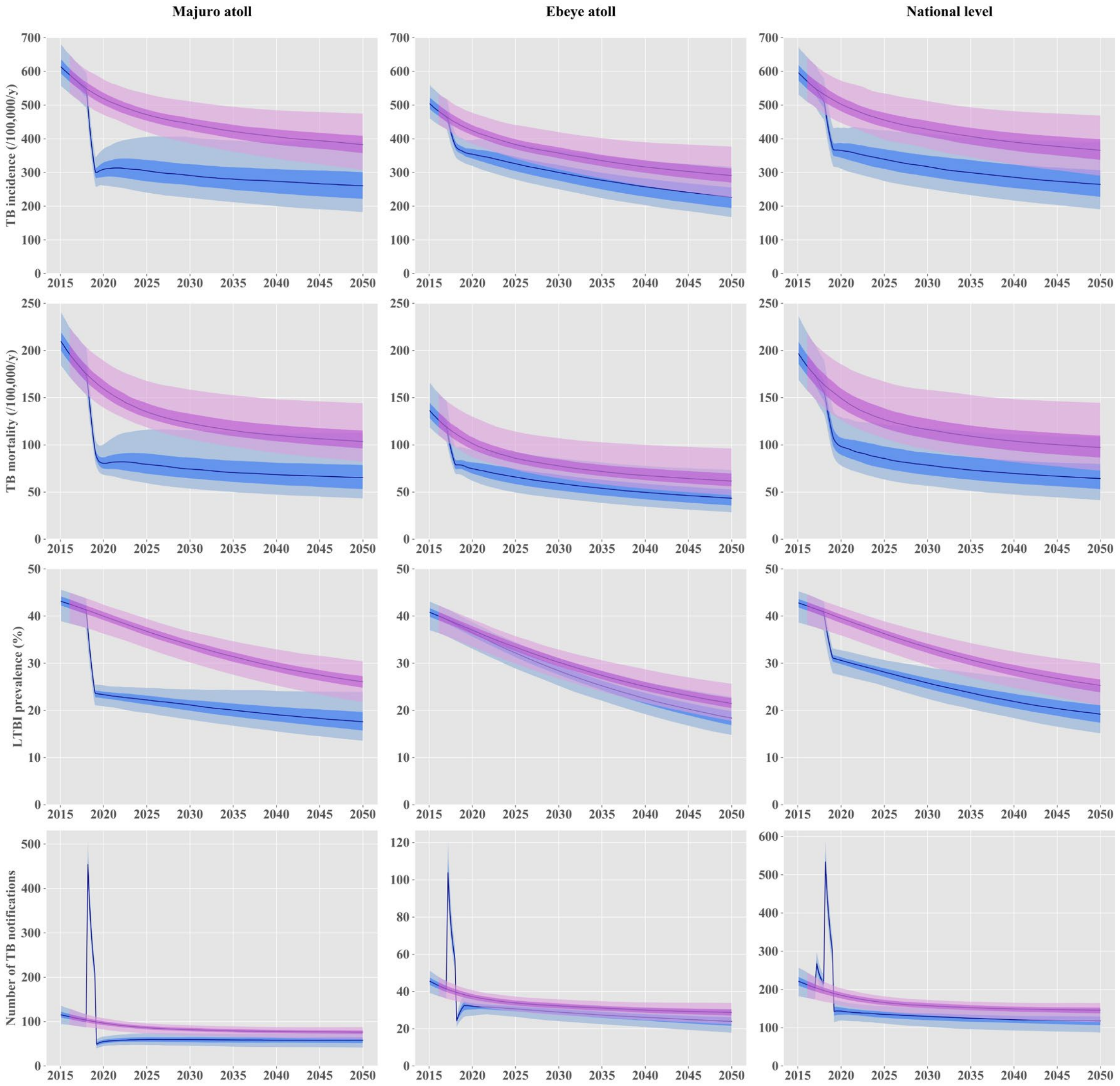

Figure S11. Projected effect of the active screening interventions implemented in 2017 and 2018, assuming increased mixing between the different islands.

The solid lines represent the median estimates. The shaded areas show the interquartile ranges (dark shade) and 95% credible intervals (light shade) projected in the absence of any intervention (pink) and under a scenario including the interventions implemented in 2017-2018 in Majuro and Ebeye (blue).

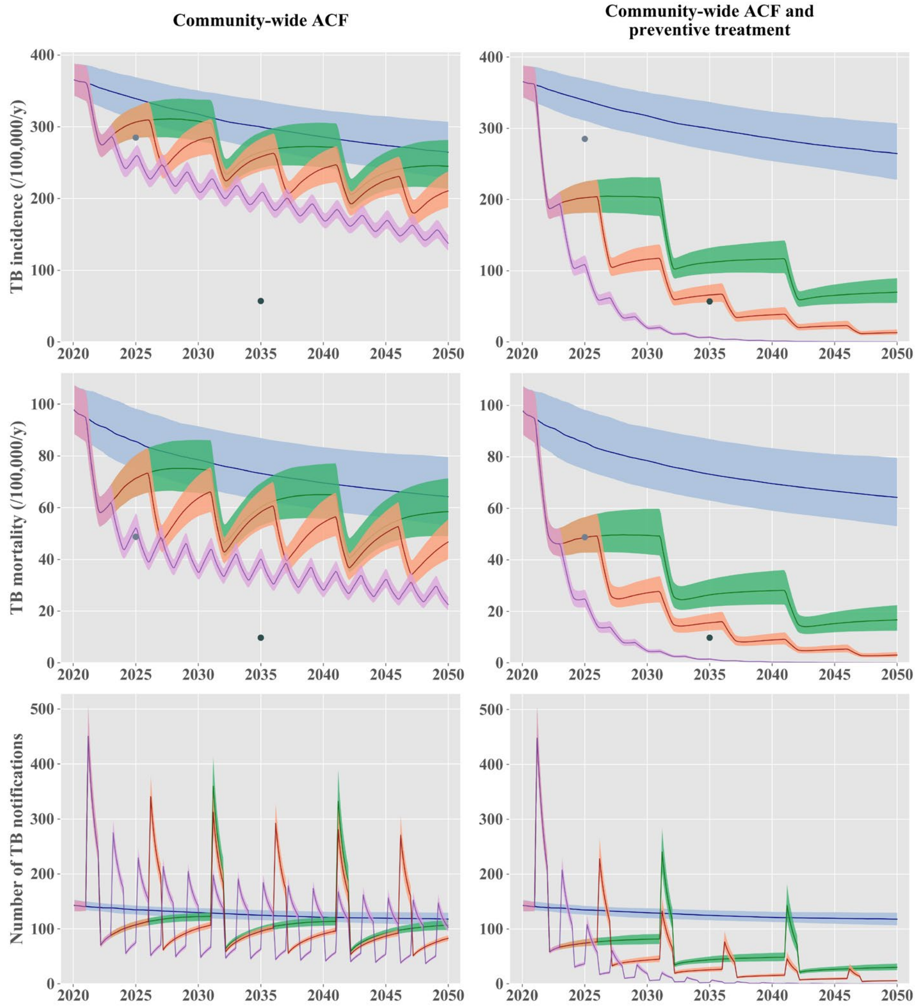

Figure S12. Projected effect of periodic community-wide interventions, assuming increased mixing between the different islands.

The solid lines represent the median estimates, and the shaded areas show the interquartile credible ranges. The “status-quo” scenario is represented in blue in all panels. The left column of panels presents scenarios including nationwide active case finding (ACF) repeated every two years (purple) or every five years (orange) or every ten years (green). The right column of panels presents nationwide ACF scenarios combined with mass latent infection screening and treatment, repeated every two years (purple) or five years (red). The light and dark grey dots show the 2025 milestones and the 2035 targets, respectively, according to the End TB Strategy.

Benefit risk of active screening interventions

Table S5 includes the same findings as Figure 6 (main text) in a tabular format.

Scenario	TB disease episodes averted by 2050	TB deaths averted by 2050	Individuals treated for LTBI by 2050	Serious adverse events by 2050
**Reference: no screening counterfactual**				
Status-quo including 2017-2018 interventions	1948 (1173-2742)	643 (370-949)	5807 (5261-6370)	93 (84-102)
**Reference: status-quo including 2017-2018 interventions**				
Screening every 10 years	2511 (1816-3534)	616 (397-993)	14354 (12498-17366)	230 (200-278)
Screening every 5 years	3187 (2293-4825)	772 (492-1362)	17205 (14862-20814)	275 (238-333)
Screening every 2 years	3793 (2714-5996)	918 (578-1622)	18391 (15869-21339)	294 (254-341)

Table S5. TB disease episodes and TB deaths averted compared to total number of preventive treatment courses and serious adverse events by 2050.
Median estimates and 95% credible intervals (in brackets). Cumulative numbers estimated between 2017 and 2050.

Projected impact of periodic interventions (log scale)

The projected epidemics under the different intervention scenarios are presented in the main text (Figure 4). Here below, the same outputs are represented on a log scale in order to visualise better whether the interventions considered could achieve the pre-elimination target by 2050.

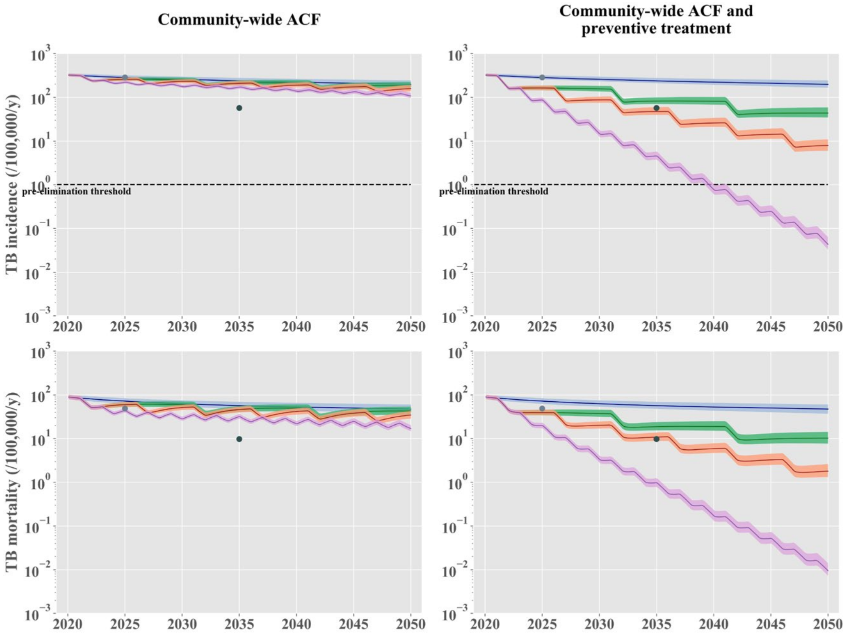

Figure S13. Projected effect of periodic community-wide interventions (log-scale).
The solid lines represent the median estimates, and the shaded areas show the interquartile ranges. The "status-quo" scenario is represented in blue in all panels. The left column presents scenarios including nationwide active case finding (ACF) repeated every two years (purple), every five years (orange), or every ten years (green). The right column presents scenarios including nationwide ACF combined with mass latent infection screening and treatment, repeated every two years (purple) or five years (red). The light and dark grey dots show the 2025 milestones and the 2035 targets, respectively, according to the End TB Strategy. The horizontal dashed lines represent the pre-elimination threshold defined by the WHO as a TB incidence of 1 per-100,000-persons-per-year.

References

1. Trauer JM, Ragonnet R, Doan TN, McBryde ES. Modular programming for tuberculosis control, the “AuTuMN” platform. BMC Infect Dis. 2017;17(1).
2. Trauer JM, Denholm JT, Waseem S, Ragonnet R, McBryde ES. Scenario analysis for programmatic tuberculosis control in Western Province, Papua New Guinea. Am J Epidemiol. 2016;183(12).
3. Doan TN, Varleva T, Zamfirova M, Tyufekchieva M, Keshelava A, Hristov K, et al. Strategic investment in tuberculosis control in the Republic of Bulgaria. Epidemiol Infect. 2019;
4. Ragonnet R, Underwood F, Doan TN, Rafai E, Trauer JM, McBryde ES. Strategic planning for tuberculosis control in the Republic of Fiji. Trop Med Infect Dis. 2019;4(2).
5. Ragonnet R, Trauer JM, Scott N, Meehan MT, Denholm JT, McBryde ES. Optimally capturing latency dynamics in models of tuberculosis transmission. Epidemics. 2017;
6. Watson CH, Coriakula J, Ngoc DTT, Flasche S, Kucharski AJ, Lau CL, et al. Social mixing in Fiji: Who-eats-with-whom contact patterns and the implications of age and ethnic heterogeneity for disease dynamics in the Pacific Islands. PLoS One [Internet]. 2017 Dec 1 [cited 2021 Oct 13];12(12):e0186911. Available from: https://journals.plos.org/plosone/article?id=10.1371/journal.pone.0186911
7. Nguipdop-Djomo P, Heldal E, Rodrigues LC, Abubakar I, Mangtani P. Duration of BCG protection against tuberculosis and change in effectiveness with time since vaccination in Norway: a retrospective population-based cohort study. Lancet Infect Dis [Internet]. 2016;16(2):219–26. Available from: http://www.ncbi.nlm.nih.gov/pubmed/26603173
8. Medical Research Council. BCG and vole bacillus vaccines in the prevention of tuberculosis in adolescence and early adult life. Bull World Heal Organ [Internet]. 1972/01/01. 1972;46(3):371–85. Available from: http://www.ncbi.nlm.nih.gov/pubmed/4537855
9. Behr MA, Warren SA, Salamon H, Hopewell PC, Ponce de Leon A, Daley CL, et al. Transmission of Mycobacterium tuberculosis from patients smear-negative for acid-fast bacilli. Lancet [Internet]. 1999;353(9151):444–9. Available from: http://www.ncbi.nlm.nih.gov/pubmed/9989714
10. Tostmann A, Kik S V, Kalisvaart NA, Sebek MM, Verver S, Boeree MJ, et al. Tuberculosis transmission by patients with smear-negative pulmonary tuberculosis in a large cohort in the Netherlands. Clin Infect Dis [Internet]. 2008;47(9):1135–42. Available from: http://www.ncbi.nlm.nih.gov/pubmed/18823268
11. Ragonnet R, Trauer JM, Geard N, Scott N, McBryde ES. Profiling Mycobacterium tuberculosis transmission and the resulting disease burden in the five highest tuberculosis burden countries. BMC Med [Internet]. 2019 Nov 22 [cited 2020 May 16];17(1):208. Available from: https://bmcmedicine.biomedcentral.com/articles/10.1186/s12916-019-1452-0
12. Ragonnet R, Flegg JA, Brilleman SL, Tiemersma EW, Melsew YA, McBryde ES, et al. Revisiting the Natural History of Pulmonary Tuberculosis: A Bayesian Estimation of Natural Recovery and Mortality Rates. Clin Infect Dis. 2020;
13. Haario H, Saksman E, Tamminen J. An adaptive Metropolis algorithm. Bernoulli. 2001;
